# Conserved G-Quadruplexes Regulate the Immediate Early Promoters of Human *Alphaherpesviruses*

**DOI:** 10.3390/molecules24132375

**Published:** 2019-06-27

**Authors:** Ilaria Frasson, Matteo Nadai, Sara N. Richter

**Affiliations:** Department of Molecular Medicine, University of Padua, via A. Gabelli 63, 35121 Padua, Italy

**Keywords:** G-quadruplex, immediate early promoters, *Alphaherpesvirinae*, *Herpesvirus*, virus

## Abstract

Human *Alphaherpesviruses* comprise three members, herpes simplex virus (HSV) 1 and 2 and varicella zoster virus (VZV). These viruses are characterized by a lytic cycle in epithelial cells and latency in the nervous system, with lifelong infections that may periodically reactivate and lead to serious complications, especially in immunocompromised patients. The mechanisms that regulate viral transcription have not been fully elucidated, but the master role of the immediate early (IE) genes has been established. G-quadruplexes are non-canonical nucleic-acid structures that control transcription, replication, and recombination in many organisms including viruses and that represent attractive antiviral targets. In this work, we investigate the presence, conservation, folding and activity of G-quadruplexes in the IE promoters of the *Alphaherpesviruses*. Our analysis shows that all IE promoters in the genome of HSV-1, HSV-2 and VZV contain fully conserved G-quadruplex forming sequences. These comprise sequences with long loops and bulges, and thus deviating from the classic G-quadruplex motifs. Moreover, their location is both on the leading and lagging strand and in some instances they contain exuberant G-tracts. Biophysical and biological analysis proved that all sequences actually fold into G-quadruplex under physiological conditions and can be further stabilized by the G-quadruplex ligand BRACO-19, with subsequent impairment of viral IE gene transcription in cells. These results help shed light on the control of viral transcription and indicate new viral targets to design drugs that impair the early steps of *Alphaherpesviruses*. In addition, they validate the significance of G-quadruplexes in the general regulation of viral cycles.

## 1. Introduction

In the last years the fact that the DNA can adopt complex secondary structures other than the double-stranded (ds) form wrapped around histones and packaged as chromatin [1] has become evident [2,3,4]. Guanine (G)-rich sequences in nucleic acids can assemble into G-quadruplex (G4) structures, comprising G-tetrads linked by loop nucleotides. Each G-tetrad is composed of four G residues connected through Hoogsteen-type hydrogen bonds. G4s are highly polymorphic structures, the topology of which depends on the strand stoichiometry and polarity, the nature and length of the loops and their location in the sequence [5]. The G4 parallel, antiparallel or mixed topology is directly correlated to the *syn* and *anti* conformational state of the glycosidic bond between the G base and the sugar [2]. The *anti* conformation characterizes a parallel folding, while antiparallel G4s can adopt both *syn* and *anti* orientations [6]. A number of different experimental techniques are used to study G-quadruplex formation, each examining different aspects of the structures, and hence reporting on different aspects of their formation. Circular Dichroism (CD) is a sensitive tool, widely used to investigate the conformations of nucleic acids. Based on the CD profile of maximum and minimum peaks at signature wavelengths (around 240, 260 and 290 nm), this technique allows identification of the G4 folding and characterization of the G4 topology [7]. UV absorption is an additional useful technique: G4s show both a hypochromic and a hyperchromic sigmoid transition at 295 and 240 nm, respectively [8]. The Thermal Difference Spectra (TDS) acquired by subtracting the UV absorbance spectra of the unfolded from the folded form of a given sample yield profiles that are characteristic of the G4 (i.e., a negative peak around 295 nm, and two positive peaks around 275 and 243 nm). Other useful techniques to highlight G4 formation are *Taq* polymerase stop assay [9] and mass spectrometry [10,11]. Finally, to acquire the structural details of G4 folding at the atomic level, NMR and X-ray crystallography are the ideal techniques, recently reported also in cells [6,12,13].

G4s have been shown to regulate key cellular processes, including gene expression and mRNA translation, many of which are in turn linked to significant human disorders [14,15,16]. The most recent literature has broadened the G4 spectrum of influence also to pathogens [17,18,19,20,21,22,23]. In the last years many viruses have been reported to have putative G4 sequences (PQS) in their genome, and in most cases the biological role of these structures and the potential administration of G4-ligands as active and selective antiviral agents have been described [24]. Interestingly, G4s and PQS that are significant from a functional standpoint have been repeatedly identified in members of the *Herpesviridae* virus family both at the genomic and mRNA level [17,18,19,25,26,27]. This family contains over 100 ds-DNA viruses that infect humans and a wide range of eukaryotic organisms, with eight members that are exclusively human pathogens [28]. These viruses differ significantly with respect to base composition and sequence arrangement of their DNA, but share many biologic properties including the ability to remain latent in their host. On the basis of these, the human non-zoonotic *Herpesviruses* have been classified into three subfamilies, i.e., *Alphaherpesvirinae*, *Betaherpesvirinae* and *Gammaherpesvirinae*. The members of the *Alphaherpesvirinae* subfamily are characterized by a short reproductive cycle, rapid spread in culture, efficient killing of infected cells, and capacity to establish latent infections primarily but not exclusively in sensory ganglia. This subfamily contains the genera *Simplexvirus* (HSV-1, HSV-2) and *Varicellovirus* (VZV). The life cycle of these genera is characterized by a coordinated and sequential cascade of expression of three temporal classes of viral genes [29,30]: the viral tegument protein VP16 binds to the TAATGARAT signal sequence and activates transcription of the immediate early (IE) viral genes [31], the products of which in turn activate transcription of the early and late viral genes; transcription of the late viral genes is also coupled to viral DNA replication. IE gene products include regulatory proteins, while early and late gene products comprise the viral replication machinery and the structural components of the virus, respectively.

We have recently demonstrated and visualized the presence of G4s in the HSV-1 genome [27] and their targeting by two classes of G4-ligands, which efficiently inhibited viral replication [17,32]. In addition, we evidenced that several members of the *Herpesviridae* family, i.e., HSV-1, HSV-2, VZV, HHV-4 and HHV-8, are statistically significantly enriched in G4 patterns [33]. These data suggest a conserved and essential role of G4s in *Herpesviruses*.

Here we show that conserved and stable G4s are present in crucial points of the *Alphaherpesvirinae* (i.e., HSV-1, HSV-2 and VZV) genome, in particular in promoters of IE genes, the major regulators of the life cycle of these viruses. The IE promoters contain multiple G4s on both leading and lagging strands; these viral G4s are in general stable and can be further stabilized by G4-ligands. Their folding results in inhibition of transcription. A significant parallelism in conserved G4s in key genomic regulatory regions within members of the same viral subfamily becomes apparent. Our results point for the first time to a G4-mediated regulation of the initial viral steps of this important class of human pathogens and may pave the way to a deeper understanding of *Alphaherpesvirinae* infection regulation.

## 2. Results

### 2.1. Detection of PQS in Herpesviridae Immediate Early (IE) Promoters

G4s have been reported to regulate transcription, both at the cellular and viral level, when embedded in promoter regions [15,24]. The HSV-1 infective cycle is predominately driven by the five immediate early (IE) genes, namely *ICP0*, *ICP4*, *ICP27* (UL54), *ICP22* and *ICP47* (US12) [34]. Our previous investigation showed that the GGG-island type PQS in HSV-1 were distributed along four defined genomic features, i.e., coding sequences (CDS), repeat regions (RR), 5′- and 3′-untranslated (UTR), and promoter regions, with a particularly high concentration in the RR and 5′–regulatory region [33]. However, while CDS and RR are explicitly described in RefSeq and GenBank databases, the annotation for promoters in viruses is generally inconsistent. To check whether G4s were reliably present in the promoters of HSV-1 IE genes, we first selected the region up to 1 kb upstream of the Transcription Start Site (TSS) of any unambiguous IE transcripts in the HSV-1 reference genome (NC_001806.2) [35]. The selected regions were examined for the presence of specific genomic features, such as the TAATGARAT signals, TATA boxes, replication origins, polyA signals or CDSs on the opposite strand, in order to restrict our analysis to sequences that most likely contained promoters. This analysis yielded five prominent sequences.

To corroborate our findings, we looked for any reported biological evidence: interestingly, all five regions were indicated to exert promoter activity in IE genes [36,37,38,39,40], with the peculiarity that the same sequence repeated in two different genomic regions constitutes the promoter of the *ICP22* and *ICP47* genes [37]. We thus checked these four promoter regions for PQS by running an initial algorithm-driven analysis to search for [G(2)L(1–7)]4, [G(3)L(1–12)]4 and [G(4)L(1–12)]4-type G4s, followed by a manual sequence revision to highlight the possible presence of “non-canonical” G4s (i.e., G(3)L(0-12)) or bulged PQS [41]. Subsequently the degree of conservation of each sequence was retrieved from the G4 virus website (http://www.medcomp.medicina.unipd.it/main_site/doku.php?id=g4virus#data_download) in order to discard non-conserved regions. Interestingly, all identified PQS were fully conserved, both in terms of G-tracts and loop-length and -composition, in the entire set of fully sequenced genomes present in the National Center for Biotechnology Information (NCBI) databank. The HSV-1 IE promoters contained multiple PQS (Table 1), embedded both in the leading and lagging strand.

**Figure 1:**
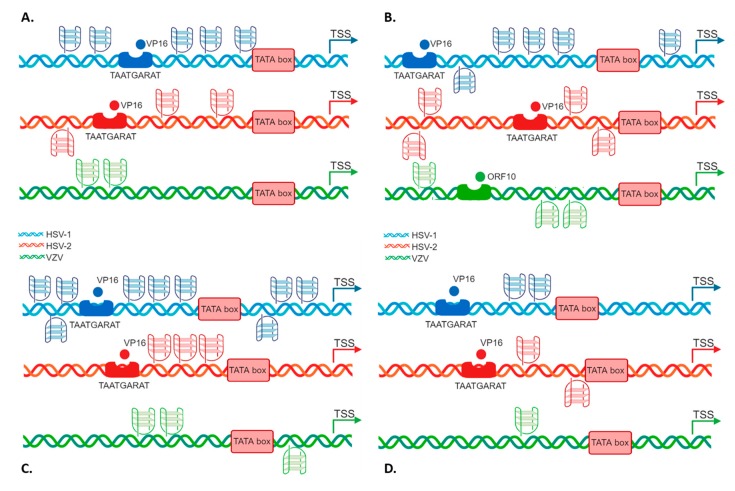
Schematic comparative representation of G4s in *Alphaherpesvirus* IE promoters. The four IE promoters (**A**. *ICP0*; **B**. *ICP4* or *ORF62*; **C**. *ICP22/ICP47* or *ORF63* and **D**. *ICP27*) are reported. HSV-1, HSV-2 and VZV promoter schemes are shown in blue, red and green, respectively. The TAATGARAT region is shown as a blue/red box with the VP16 protein nearby (or as a green box with *ORF10* protein in the case of VZV); the TATA box is represented as a red box; the arrow indicates the position of the Transcriptional Start Site (TSS). The position of each of these features on the double helix with respect to the TSS is on scale. In each promoter, the predicted G4s are shown above and below the DNA strand for G4s present in the leading and lagging strand, respectively.

*ICP0* displayed five PQSs, two preceding the TAATGARAT signal, and three upstream the TATA box, all on the leading strand (Figure 1A); *ICP4* promoter contained five PQSs, four embedded in the region encompassing the TAATGARAT signal and the TATA box, the most distal G4 being on the lagging strand, and one close to the TSS (Figure 1B); *ICP22/ICP47* promoter included 9 PQSs, 6 upstream of the TATA box signal, both on the leading and lagging strand and the remaining 3 between the TATA box and the TSS (Figure 1C); *ICP27* promoter displayed two partially overlapped PQSs embedded between the TAATGARAT signal and the TATA box (Figure 1D). Notably, *ICP0* and *ICP22/ICP47* promoters contained bulged PQS. Moreover, the majority of the predicted PQS had at least one region possibly forming GC stem loops.

To check whether the G4 pattern identified in the HSV-1 IE promoters was also present and conserved in all members of the *Alphaherpesviruses*, we extended our analysis to HSV-2 and VZV IE gene promoters. First of all, we identified the homologues genes in the three viruses (Table 2) [42,43] and then performed the same algorithm and manual predictive analysis described for HSV-1.

Notably, also HSV-2 and VZV presented fully conserved PQS within the IE promoters, as reported in Table 3.

**Figure 2:**
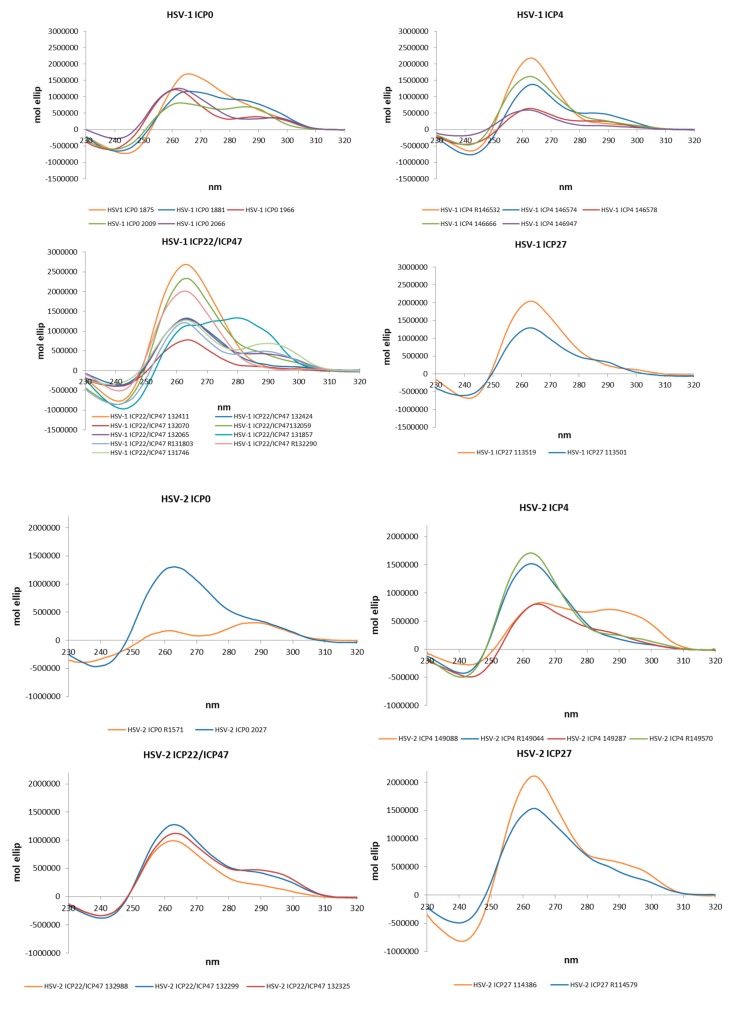

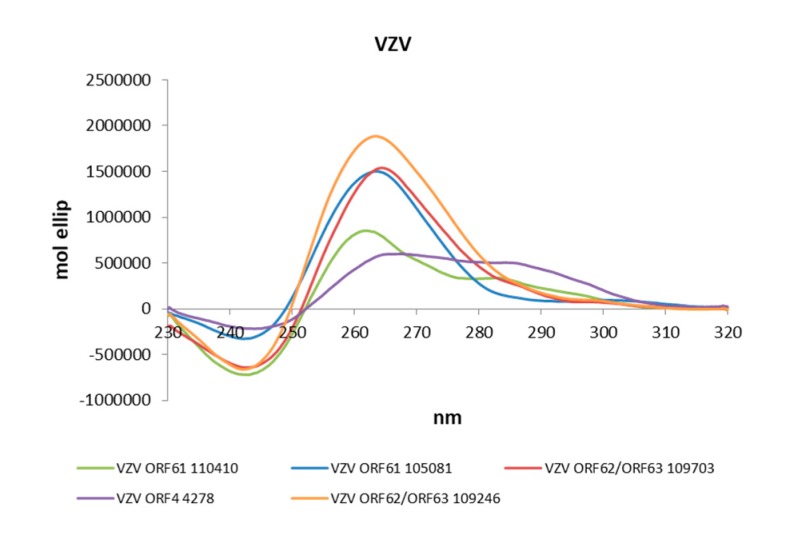
Circular Dichroism (CD) spectra of the PQS embedded in the *Alphaherpesvirus* IE promoters in PB 20 mM and KCl 70 mM.

HSV-2, has always been considered a close homologue of HSV-1, but recent research highlighted that the two viruses are less related than initially believed [44]. Our analysis retrieved fewer PQS in the HSV-2 IE promoter regions with respect to HSV-1, but all of them were conserved in all sequenced circulating strains. VZV, despite sharing a similar biology with HSV-1 and HSV-2, including latency in sensory neurons, has a different genome organization and gene expression regulation [45,46]. The number of PQS predicted for VZV promoters was the smallest among the three viruses (*n* = 6); however, all of them retained the highest degree of conservation. PQS were retrieved on both strands: similarly to HSV-1 *ICP22* and *ICP47*, *ORF62* and *ORF63* of VZV are transcribed in opposite directions from a 1.5-kb intergenic region that acts as a bidirectional promoter [44] and also contains the VZV origin of replication [47]. *ORF62* and *ORF63* TSSs lie on opposite strands, thus PQS on the leading strand with respect to *ORF63*, on the lagging strand may act as regulators of *ORF62*, and vice versa. VZV is also the only virus with a predicted GG-island type PQS (*ORF4* 4278). Even if the TAATGARAT sequence is not present in VZV promoters, a TAATGARAT-VP16-like transcriptional enhancement can be mimicked by the ORF10 protein [48]. The ORF10 protein has been shown to influence the transcription of the VZV immediate-early (IE) gene, *ORF62*, but not of *ORF61*, *ORF4* or *ORF63* [49,50]. The relative comparison of PQS position on each *Alphaherpesviruses* IE promoter is reported in Figure 2.

These data indicate the possibility that IE gene expression of *Alphaherpesviruses* is regulated by G4s.

### 2.2. The Identified PQSs Fold into G4 Structures

The actual ability of *Alphaherpesvirus* IE promoter PQSs to form G4 structures was initially assessed by Circular Dichroism (CD), as this technique is considered a reliable tool to study nucleic acids conformation [51]. All sequences were analysed in the presence of K^+^ to establish G4 folding, topology and thermal stability. Table 4 and in Figure 2 show that all predicted PQS did actually fold into G4 structures. The large majority (77%) of G4s displayed a parallel topology, with a maximum positive peak at ∼260 nm and a minimum at ∼240 nm. Some G4s were also characterized by a modest shoulder at 295 nm, without wavelength shift of the minimum peak at 240 nm and were considered parallel G4s (Table 4). The remaining sequences (18%) showed two maxima at 265 and 295 nm and a minimum at 245 nm and were thus classified as hybrid structures.

The stability of all G4s was assessed by melting experiments monitored by CD. The melting temperatures (T_m_) were calculated according to the van ’t Hoff equation (Table 4 and Figure S1). In half of the analysed sequences (58%) the CD signal decreased over temperature, leading to discrete T_m_ values. Notably, the remaining sequences (42%) maintained their G4 folding up to the maximum tested temperature (90 °C). For hybrid structures, where two transitions were visible, T_m_ values were calculated at both wavelengths.

We next investigated the *Alphaherpesvirus* IE promoter sequences in the presence of the commercially available G4-ligand, BRACO-19 (B19), which has been reported to specifically recognize and stabilize G4 structures over double- and single-stranded nucleic acids [52]. The effect of B19 was assessed by CD thermal unfolding analysis. B19 was able to further stabilize all G4s, with an increase in T_m_ higher than 10 °C (Table 4). In cases where several transitions were observed, T_m_ values for each transition were annotated. The results obtained with B19 further confirm the ability of all the predicted sequences to fold into G4 structures.

TDS spectra analysis was next employed to corroborate G4 folding. Mergny and others [8,53] established that G4 structures exhibit a TDS negative peak at 295 nm, and two positive peaks at 275 and 243 nm, respectively. The vast majority (86%) of analysed sequences displayed the G4 distinctive TDS spectrum (Table 4 and Figure S1). Bulged sequences, sequences containing more than four G-islands and sequences retaining the ability to form stem-loops displayed the characteristic TDS at higher cation concentration (KCl 150 mM), which allowed increased stabilization of the G4 over possible competitor structures. Five sequences displayed only the two positive peaks, and thus reported a non-definite TDS spectrum (indicated as +/−). It is possible that “non canonical” G4 structures cannot confidently be distinguished by TDS analysis.

**Figure 3:**
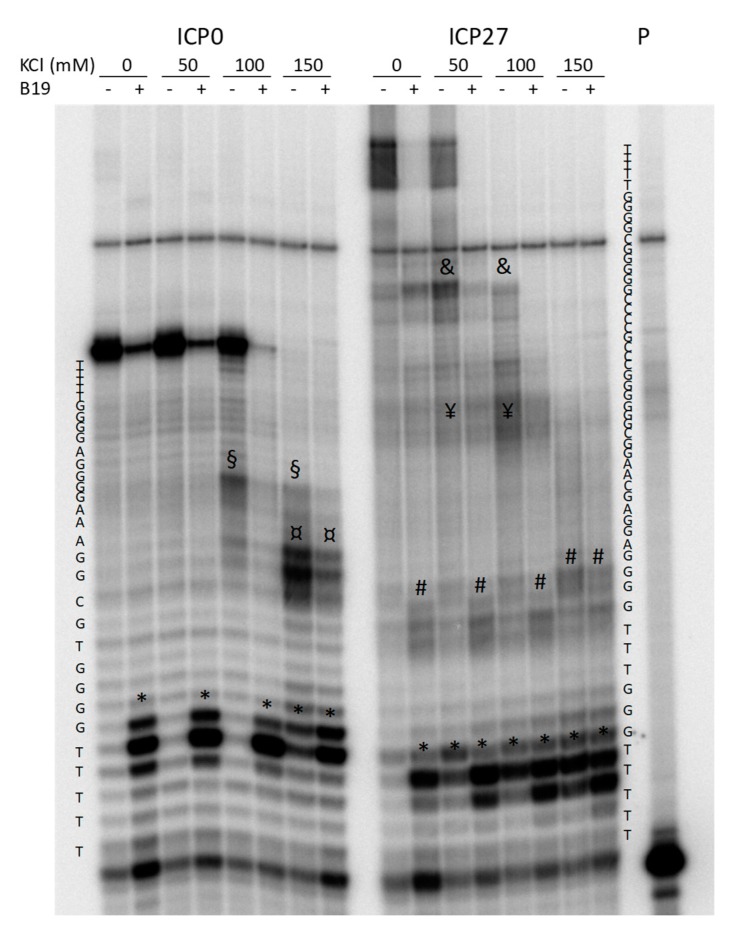
Sequencing PAGE of *Taq*-amplified *ICP0* and *ICP27* templates in the absence or presence of increasing concentrations of KCl and the G4-ligand B19. Symbols *, ¤, §, #, ¥ and & indicate pausing sites just before the G4 regions in the templates. P indicates the lane of the labeled primer.

For these reasons, two non-canonical G4s were additionally explored by *Taq* polymerase stop assay. These corresponded to the HSV-1 *ICP0* and *ICP27* promoter sequences (*ICP0* 2066 and *ICP27* 113501+113519): the first one was a putative bulged G4 that displayed G4-representative CD and TDS profiles, the second with long G-tracts and the possibility to form GC stem loops, had showed an ambiguous TDS profile. The *Taq* polymerase stop assay indicates the ability of a G4 to block or pause the enzyme activity and allows evaluation of G4 structures within an extended DNA environment. Samples were incubated in the absence and presence of increasing concentrations of KCl (Figure 3), and 1 µM B19. Stop sites resulted specific and located mainly at, or just before, the most 3′ G-tract involved in G4 formation (Figure 3, * symbol). Additional stop sites (Figure 3, ¤, §, #, ¥ and & symbols) corresponding to other G-tracts indicated the “breathing” of the G4 structure and possibly the ability of the tested oligonucleotides to fold into multiple G4 structures, preferentially stabilized at different K^+^ concentrations. The G4-ligand B19 blocked enzyme processing mainly in correspondence of the most 3′ tract involved in G4 formation, suggesting that the structure with the longest sequence is likely the most stable and thus the one preferentially bound by B19 (Figure 3, * symbol). These data indicate that both the tested non-canonical G4s are able to fold into G4, and thus provide evidence that the TDS assay may lack the negative peak at 295 nm while still depicting a non-canonical G4.

Taken together these data indicate that IE PQS can fold into dynamic G4 structures, which are induced and stabilized by increasing concentrations of K^+^ and a G4-ligand.

### 2.3. G4s Tune IE Promoter Activity at the Cellular Level

**Figure 4:**
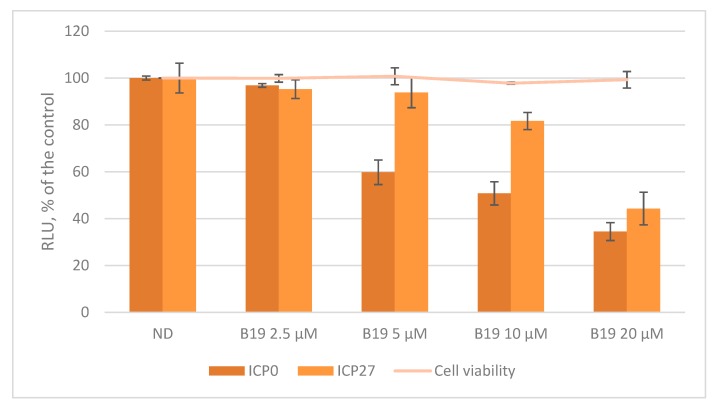
Luciferase assay on HSV-1 ICP0 and ICP27 promoters in U-2 OS (human osteosarcoma) cells in the presence of the G4 ligand B19 (2.5–20 µM). Cell viability, determined by MTT (3-(4,5-dimethylthiazolyl-2)-2,5-diphenyltetrazolium bromide) assay, in the presence of B19 is also indicated.

The biological role of G4s at the promoter level is still matter of debate since little is known on how G4 structures and their interaction with transcription factors and other proteins may contribute to regulate viral promoters. Here, we decided to study the activity of two representative promoters, i.e., HSV-1 *ICP0* and *ICP27* in cells. These two promoters were chosen both for their peculiar G4 content and for their unique roles during HSV-1 infection. ICP0 is the viral ubiquitin ligase that has been described to act as a powerful viral transactivator—both during productive infection and in reactivation from latency [54]. In contrast, at the nuclear level ICP27 induces the expression of a restricted number of early and late genes, such as UL42 (polymerase processivity factor) and UL44 (glycoprotein C) but it remains highly expressed during the whole viral life cycle [55]. The two sequences, *ICP0* and *ICP27* (NC_001806.2 nts 1551-2261 and nts 113451-113734, respectively) were cloned upstream of the firefly luciferase gene in a promoterless plasmid. The two promoters were studied in U-2 OS cells, a human osteosarcoma cell line that withstands HSV-1 infection [56], in the absence and presence of increasing concentrations of B19. The assay was performed monitoring also cell viability in the presence of B19 (CC_50 U2-OS_ > 100 µM) to avoid luciferase signal variation due to cytotoxicity. As shown in Figure 4, the activity of both promoters decreased in a dose dependent manner, up to ~65% of the untreated control in *ICP0* and up to ~55% in *ICP27*, proving that in this case G4s act as suppressors of transcription.

These findings support the hypothesis that G4s tune the activity of *Alphaherpesvirus* IE promoters, as indicated by the B19-mediated G4 stabilization, which down-regulates ICP0 and ICP27 transcription.

## 3. Discussion

In the past few years, interest in the characterization of G4 structures and their role within viral genomes has greatly increased, providing possibly innovative antiviral targets against many human pathogens. In this context, our group proved that HIV-1 transcription is modulated by the tuned folding/unfolding of G4s located in the U3 region of the LTR promoter [23,57]. We successively demonstrated that the HSV-1 genome contains an impressively high number of putative G4s that were visualized during infection in cells by means of an anti-G4 antibody [17,27]. In support of the presence and involvement of G4s in key viral processes, we found that two G4-ligands (B19 and a core extended naphthalene diimide) displayed remarkable antiviral activity in both viruses [17,32,58,59].

The HSV-1 cycle is strictly regulated by five IE proteins (namely ICP0, ICP4, ICP22, ICP27 and ICP47), that are also responsible in vivo for the establishment of latency at the neuronal level [34,60]. The five genes that encode for these proteins are immediately transcribed after infection and mutations altering their expression are strongly associated with a dysregulated viral cycle [60].

In this work, we investigated the presence of G4s within the promoter regions of IE genes of all members of the *Alphaherpesvirus* subfamily. The promoters were unambiguously identified based both on the presence of characterizing motifs, such as the TATA box and the TAATGARAT sequence and on previously reported biological evidence. In each promoter, we found several multiple and non-overlapping G4 folding motifs, interestingly located both on the leading and lagging strands. At the promoter level, G4 structures on the lagging strand have been shown so far only in few examples, in particular in the promoter region of the REarranged during Transfection (RET) proto-oncogene [61,62] and as regulators of DNA strand replication [63] in mammalian cells. In the human papillomavirus as well as in the human cytomegalovirus, G4s on the lagging strand were predicted only in non-coding regulatory regions, lacking experimentally proved promoter activity [18,64]. Promoters containing multiple G4s have been described in oncogenes [65,66,67], however, none of these features has been reported in viruses as yet. This work indicates the possibility that the large and strictly regulated genome of *Herpesviruses* controls gene transcription at the promoter level through G4-mediated mechanisms, as reported for the human genome. ICP4 is the major viral transcription factor, and its promoter presents 5 G4s. The G4 most distant from the TSS is embedded in the lagging strand close to the TAATGARAT sequence, which is a recognition signal for the viral protein VP16 [31]: it is possible that this G4 is involved in the transactivation activity of VP16. The *ICP22* and *ICP47* promoters, which share the same sequence albeit in two different locations along the genome, display a high number of G4s, also in this case located on both strands: we envisage the possibility of a control on both transcription and replication, since in the same region resides oriS, one of the origins of viral genome replication [68]. ICP0 plays a major role both on viral transactivation during the lytic cycle and in reactivation from latency: it contains five G4s nearby the TAATGARAT sequence and TATA box. ICP27 is an essential HSV-1 IE protein along with ICP4, but in contrast to the latter which is expressed only in the early stages and is involved in transcription initiation, ICP27 is a multifunctional protein and its expression remains high throughout the viral cycle, with post-translational modifications that regulate its activity [69,70,71]. The *ICP27* promoter is the one that contains fewer G4s. Four out of five *ICP0* G4s, displaying low Tm (50 < Tm < 68 °C), were significantly stabilized by the G4-ligand, whereas *ICP27* G4s were very stable even in the absence of B19 and thus were less affected by the G4-ligand. These features are likely the reason why we observed a lower degree of response to the G4-ligand B19 in cells for *ICP27* compared to *ICP0*.

As HSV-1, HSV-2 and VZV are closely related viruses, all displaying a wide host-cell range, similar pathogenesis and analogous genome organization, we evaluated the presence of G4s also in HSV-2 and VZV IE promoters. The vast majority of the retrieved sequences, besides being fully conserved within all sequenced strains, were experimentally proven by at least two techniques to fold into G4 structures.

Finally, we found that many of the *Alphaherpesvirus* IE promoter G4-forming sequences described in this work formed “non-canonical” G4s: they were able to fold even in the absence of loops (i.e., HSV-1 *ICP4* 149570; VZV *ORF62/63* 109246) or in the presence of putative stem-forming sequences in the loop (i.e., HSV-1 *ICP0* 1966; HSV-1 *ICP4* 146666, 146578 and 146947; HSV-1 *ICP22/ICP47* R131803, 131857, 132059) or with bulges (i.e., HSV-1 *ICP0* 2009 and 2066; HSV-2 *ICP4* 149044, 149287). This fact indicates that the classic prediction methods based on motif recognition may overlook many non-canonical G4s that actually form. In this view, the newest machine learning models trained with sequences of experimentally validated G4 may yield more reliable results [72]. Moreover, several G4s (see HSV-1 *ICP4*, HSV-1 *ICP22/ICP47*, HSV-2 *ICP0*, HSV-2 *ICP27* and VZV *ORF62/ORF63*) presented 5 G-tracts; multiple G-tracts have been previously described for another strictly regulated viral promoter, the LTR sequence of HIV-1 virus [23]. These data may corroborate the recently proposed hypothesis by Burrows CJ and co-workers that the tracts exceeding four work as ”spare tires” in promoters [73].

The described data provide a possible new direction for active antiviral design. Up to date, only polymerase inhibitors have been found active against HSV-1, HSV-2 and VZV. However, because of the emergence of resistant strains that may be extremely dangerous especially in transplant and immunocompromised patients [74], new antivirals with a different mechanism of action are highly wished for. We propose that selective anti-IE promoters G4-ligands could hinder the viral cycle at a much earlier stage, preventing the discomfort, the possible neuronal damage, the painful sequelae (specific to VZV) in normal human hosts and the possible life-threatening effects in immunocompromised patients caused by the *Alphaherpesviruses*.

## 4. Materials and Methods

### 4.1. PQS Detection and Evaluation of Conservation

The complete set of human *Alphaherpesvirus* genome sequences were downloaded from GenBank. The first analysis was performed with free G4 hunting software (QGRS http://bioinformatics.ramapo.edu/QGRS/index.php and Quadbase http://quadbase.igib.res.in/), using the standard parameters of the two programs (Minimum G-tetrad 2 and 3, Loop length1-7 for GG and 1–12 for GGG). Since *Herpesviridae* are dsDNA viruses, the presence of PQS was analysed on both genome strands.

### 4.2. Oligonucleotides and Cell Lines

Desalted oligonucleotides were purchased from Invitrogen and from Sigma-Aldrich, Milan, Italy (Table 1, Table 3). U2-OS (ECACC 92022711) were purchased from Sigma Aldrich and maintained in Dulbecco’s Modified Eagle’s medium (DMEM) (Gibco, Thermo Fisher Scientific, Waltham, MA, USA) supplemented with 10% heat-in- activated fetal bovine serum (FBS, Gibco, Thermo Fisher Scientific, Waltham, MA, USA). Cells were grown in a humidified incubator maintained at 37 °C with 5% CO_2_.

### 4.3. Circular Dichroism Spectroscopy

DNA oligonucleotides were diluted to a final concentration (4 μM) in phosphate buffer (PB, 20 mM, pH 7.4) and KCl 70 mM. All samples were annealed at 95 °C for 5 min and gradually cooled to room temperature. The G4-ligand Braco-19 (B19, ENDOTHERM, Saarbruecken, Germany) was added from stock at final concentration of 16 µM. CD spectra were recorded on a Chirascan-Plus (Applied Photophysics, Leatherhead, UK) equipped with a Peltier temperature controller using a quartz cell of 5 mm optical path length, over a wavelength range of 230–320 nm. For the determination of T_m_, spectra were recorded over a temperature range of 20–90 °C, with temperature increase of 5 °C. The reported spectra are baseline-corrected for signal contributions due to the buffer. Observed ellipticities were converted to mean residue ellipticity (θ) = deg × cm^2^ × dmol^−1^ (mol ellip). T_m_ values were calculated according to the van’t Hoff equation, applied for a two-state transition from a folded to unfolded state, assuming that the heat capacity of the folded and unfolded states are equal [75].

### 4.4. Taq Polymerase Stop Assay

*Taq* polymerase stop assay was carried out as previously described [23]. Briefly, the 5′-end labelled primer was annealed to its template (Table S1) in lithium cacodylate buffer in the presence or absence of KCl (50–150 mM) and by heating at 95 °C for 5 min and gradually cooling to room temperature. Where specified, samples were incubated with 1 µM B19 for 24 h after the annealing step. Primer extension was conducted with 2 U of AmpliTaq Gold DNA polymerase (Applied Biosystem, Carlsbad, California, USA) at 47 °C for 30 min. Reactions were stopped by ethanol precipitation, primer extension products were separated on a 16% denaturing gel, and finally visualized by phosphorimaging (Typhoon FLA 9000).

### 4.5. Plasmids Construction

The ICP0 and ICP27 promoter regions were amplified by PCR on the HSV-1 genome (GU734771.1) extracted from U2-OS infected cells. The promoter amplicons were subcloned into pGL4.10-Luc2 (Promega) within XhoI and *Hin*dIII sites. The resulting pGL4.10-ICP0 and pGL4.10-ICP27 vectors contained the sequenced regions corresponding to nts 1551-2261 (ICP0) and nts 113451-113734 (ICP27) in the HSV-1 reference genome (NC_001806.2), fused to the luciferase-coding region.

### 4.6. Reporter Assays

Vectors pGL4.10-ICP0 and pGL4.10-ICP27 (150 ng each) were transfected in 1.2 × 10^5^ U2-OS cells per well onto 12-well plates, using *Lipo3000* transfection reagent (Invitrogen, Life Technologies Italia, Monza, Italy). B19 was added to the cell medium 1 h after transfection at increasing concentrations (5–20 µM), to avoid interference, if any, with transfection. Expression of firefly luciferase was determined 24 h after transfection using the Britelite plus Reporter Gene Assay System (PerkinElmer Inc., Milan, Italy) at a Victor X2 multilabel plate reader (PerkinElmer Inc., Milan, Italy), according to the manufacturer’s instructions. Cells were lysed in 0.1% Triton-X100-PBS and protein concentration was determined by BCA assay (Thermo Scientific Pierce, Monza, Italy). Luciferase signals were subsequently normalized to total protein content, according to the manufacturer’s protocol (http://ita.promega.com/~/pdf/resources/pubhub/cellnotes/normalizing-genetic-reporter-assays/). Each assay was performed in duplicate and each set of experiments was repeated at least three times.

### 4.7. Cellular Cytotoxicity

Cytotoxic effects were determined by MTT assay. U-2 OS cells were grown and maintained according to manufacturer’s instructions (https://www.lgcstandards-atcc.org). Cells were plated into 96-microwell plates to a final volume of 100 μL and allowed an overnight period for attachment. The following day, the tested compound (B19) was added to each well and tested in triplicate. Control cells were treated in the exact same conditions. Cell survival was evaluated by MTT assay, 24 h after treatment: 10 μL of freshly dissolved solution of MTT (5 mg/mL in PBS) were added to each well, and after 4 h of incubation, MTT crystals were solubilized in solubilization solution (10% sodium dodecyl sulphate (SDS) and 0.01 M HCl). After overnight incubation at 37 °C, absorbance was read at 540 nm. Data were expressed as mean values of at least three individual experiments conducted in triplicate. The percentage of cell survival was calculated as follows: cell survival = (Awell − Ablank)/(Acontrol − Ablank) × 100, where blank denotes the medium without cells. Each experiment was repeated at least three times.

## 5. Conclusions

The work presented here provides for the first time a comprehensive analysis on the presence of G4s in the IE promoter regions of the three *Alphaherpesviruses* that infect humans. In these viruses, regulation of gene expression has been largely debated in the last decades but clear data are still lacking. Thus, the fact that in all *Alphaherpesviruses* the promoters of all genes involved in the first steps of infection and in the control of expression of the later genes contain 100% conserved G4 forming regions is a potent indication of their significance. Our data broaden the boosting recognition of G4s as molecular switches of gene expression at the viral level [24]. The in-depth understanding of the role of G4s at the viral promoter level will likely allow unravelling the mechanisms that regulate *Alphaherpesviruses* infection and latency in the human nervous system, with the subsequent possibility to design innovative drugs to manage the infection of some of the most widespread latency-associated human pathogens.

## Figures and Tables

**Table 1:** Putative Quadruplex Sequences (PQS) in the Herpes Simplex Virus 1 (HSV-1) Immediate Early (IE) promoter sequences. Each PQS is indicated by the 5′ nucleotide (nt) position on the viral reference genome (NC_001806.2). The leading and the lagging strands are indicated with respect to the Transcription Start Site (TSS) position. The conservation ratio (C (%)) of the entire sequence (G tracts and loops) among all the strains available in the National Center for Biotechnology Information (NCBI) databank is also indicated.

HSV-1	Leading Strand	Lagging Strand	C (%)
***ICP0***			
1875	GCGGGAGGGGCATGCTAATGGGGTTCTTTGGGG		100
1881	GGGGCATGCTAATGGGGTTCTTTGGGGGACACCGGG		100
1966	GGGGGCGCCGGGTTGGTCCCCGGGGACGGGG		100
2009	GGGCCTGCCTCCCCTGGGACGCGCGGCCATTGGGGG		100
2066	GGGGAGGGGAAAGGCGTGGGG		100
***ICP4***			
R146532		GGGCGGGGCGCGAGGGCGGGTGGG	100
146574	GGGCGGGGCCGGGGGTTCGACCAACGGG		100
146578	GGGGCCGGGGGTTCGACCAACGGGCCGCGGCCACGGG		100
146666	GGGGTGGGCCCGCCGGGGGGGCGGGGGG		100
146947	GGGGCCGGGGGTTCGACCAACGGGCCGCGGCCACGGG		100
***ICP22/ICP47***			
131746	GGGCAGGGGGCGGGGCCCGGG		100
R131803		GGGCGGGACCGGGGGGCCCGGGGACGGCCAACGGGCGCGCGGGG	100
131857	GGGACCAACGGGACGGCGGGCGGCCCAAGGG		100
132059	GGGGGCGGGCCCGGGCGGCGGGGGGCGGGTCTCTCCGGCG		100
132065	GGGCCCGGGCGGCGGGGGGCGGG		100
132070	GGGCGGCGGGGGGCGGGTCTCTCCGGCG		100
R132290		GGGTGGGGTGGGCGGG	100
132411	GGGGGCGGAGGAGGGGGGACGCGGGGGCGGAGGAGGGGG		100
132424	GGGGGGACGCGGGGGCGGAGGAGGGGGGACGCGGGGG		100
***ICP27***			
113501	GGGGCGGGGGCCCCGCCCGGGGGGCGG		100
113519	GGGGGGCGGAACGAGGAGGGGTTTGGG		100

**Table 2:** IE genes/proteins (they maintain the same name) in the three *Alphaherpesviruses*. The proteins of each single virus can complement the relative homologue of the other two classes. The only exception is Varicella Zoster Virus-Open Reading Frame 4 (VZV-ORF4), which cannot entirely substitute for HSV-1/HSV-2 ICP27 protein.

	HSV-1	HSV-2	VZV
	ICP0	ICP0	ORF61
	ICP4	ICP4	ORF62
**IE genes/proteins**	ICP27	ICP27	ORF4
	ICP47	ICP47	n.p.
	ICP22	ICP22	ORF63

**Table 3:** PQS in the HSV-2 and VZV IE promoter sequences. Each PQS is indicated by the 5′ nt position on the viral reference genome (NC_001798.2 and NC_001348.1). The leading and the lagging strands are indicated with respect to the TSS position. The conservation ratio (C (%)) of the entire sequence (G tracts and loops) among all the strains available in the NCBI databank is also indicated.

HSV-2	Leading Strand	Lagging Strand	C (%)
***ICP0***			
1571		GGGAAGCCGGCGCGGGGCGGTCGCCGGGGCGGAGTCCGGG	100
1913	GGGGGCGGGCACCACTCAGGGCCGCGCCGGCGGGGCGCCGGGGGG		100
2027	GGGGACGGGGCCGCCCCGAGAGGGGGGG		100
***ICP4***			
149044		GGGGCGCGCGGGGCGGGGGG	100
149088	GGGGCCGGCGGGGGCCAACGGGAGCGCGGGG		100
149287	GCGGACGCGCGGGCGTCGGGGCGGGG		100
149570		GGGGCGGCAGTGGGGGGGGGTGG	100
***ICP22/ICP47***			
132299	GGGGGGCCGGGCCGGGGGGACGGG		100
132325	GGGGGGACGGGCCGGGGGGACGGG		100
132988	GGGCCCGGACGGGGGGCGGG		100
***ICP27***			
114386	GGGGACGGCGGGGGCGGGGGCGGTGACGCCCGACGGGGAGGG		100
114579		GGGGCTGGGATGGCGGGTGTCCTCCGAGGGGG	100
**VZV**	**Leading Strand**	**Lagging Strand**	**C (%)**
***ORF61***			
104021		GGGGTCCGCCGGGCGCCCAGAAACCGGGGGGGGGTTATTTTCGGGGGGGGG	100
105081	GGGCGGGCGACGGGCGGG		100
***ORF62/ORF63***			
109246	GGGGAGGAAATATGCGGTCGAGGGGGGGG		99
109703	GCGGTTTTATGGGGTGTGGGCGGG		
110410		GGGTAAAATGGCAATGGGGGATTCCGGGGCGGGAGACCTTCGATTGGG	100
***ORF4***			
4278	GGGTGCAGGTAAGCTTGTTTGGGG		100

**Table 4:** CD and Thermal Difference Spectra (TDS) analysis of PQS embedded in the *Alphaherpesvirus* IE promoters. Both analyses were performed at 100 mM KCl. Topology and T_m_ values in the presence and absence of B19 are reported. For mixed structures, T_m_ values were calculated at both 260 and 290 nm. n.a.: oligonucleotide not available due to its too high G content.

HSV-1	Topology	T_m_	T_m_ (B19)	ΔT_m_	TDS
***ICP0***					
1875	parallel	60.9 ± 0.1	73.3 ± 0.2	12.4	+
1881	hybrid	51.9 ± 0.3/45.9 ± 0.2	80.7 ± 1.2/66.3 ± 0.5	28.8/20.4	+ *
1966	parallel	68.6 ± 0.1	>90	>21.4	+
2009	hybrid	54.9 ± 0.6/65.0 ± 0.4	69.2 ± 0.6/>90	14.3/>25	+/−
2066	parallel	>90			+
***ICP4***					
R146532	parallel	79.5 ± 0.1	>90	>10.5	+
146574	parallel	63.4 ± 0.2	>90	>26.6	+
146578	parallel	66.2 ± 0.1	80.3 ± 0.4	14.1	+/−
146666	parallel	>90			+
146947	parallel	69.2 ± 0.2	>90	>20.8	+
***ICP22/ICP47***					
131746	hybrid	>90/71.4 ± 0.8			+
R131803	hybrid	73.7 ± 0.8/76.7 ± 0.9	>90/85.1 ± 0.8	>16.3/8.4	+ *
131857	hybrid	62.4 ± 0.5/65.3 ± 0.2	69.6 ± 0.9/67.6 ± 0.4	7.2/2.3	+ *
132059	parallel	>90			+
132065	hybrid	>90/78.8 ± 0.7			+
132070	parallel	74.2 ± 0.1	>90	>15.8	+ *
R132290	parallel	>90			+ **
132411	parallel	>90			+
132424	parallel	>90			+ **
***ICP27***					
113501	parallel	>90			+/−
113519	parallel	>90			+
**HSV-2**					
***ICP0***					
1571	hybrid	77.7 ± 0.8/79.0 ± 0.7	>90/71.2 ± 1.1	>12.3/−7.8	+/−
1913	n.a.				
2027	parallel	>90			+
***ICP4***					
149044	parallel	>90			+
149088	hybrid	67.2 ± 0.7/72.6 ± 0.7	64.5 ± 0.9/>90	−2.7/>17.4	+
149287	parallel	67.2 ± 0.7	84.1 ± 2.2	16.9	+
149570	parallel	81.8 ± 0.8	>90	>8.2	+
***ICP22/ICP47***					
132299	parallel	>90			+
132988	parallel	>90			+
132325	parallel	>90			+
***ICP27***					
114386	parallel	75.9 ± 0.4	>90	>14.1	+ *
114579	parallel	66.8 ± 0.9	83.4 ± 0.7	16.6	+
**VZV**					
***ORF61***					
104021	n.a.				
105081	parallel	75.9 ± 0.4	>90	>14.1	+
***ORF62/ORF63***					
109246	parallel	>90			+
109703	parallel	57.7 ± 0.2	84.7 ± 0.5	27.0	+
110410	parallel	56.5 ± 0.4	85.2 ± 0.8	28.7	+/−
***ORF4***					
4278	hybrid	57.5 ± 0.3/53.2 ± 0.5	79.2 ± 0.9/82.0 ± 0.9	21.7/28.8	+

+/− G4 TDS lacking negative peak at 295 nm; * 150 mM KCl; ** 50 mM KCl.

## Supplementary Materials

The following are available online, Figure S1: CD thermal unfolding spectra, CD thermal unfolding fitting and TDS of *Alphaherpesvirus* IE promoter G4 sequences, Table S1: Oligonucleotides used in the *Taq* polymerase stop assay.
