# Supplementary material for: Conserved G-Quadruplexes Regulate the Immediate Early Promoters of Human Alphaherpesviruses
Source: Molecules. 2019 Jun 27;24(13):2375. doi: 10.3390/molecules24132375 (PMC6651000; doi:10.3390/molecules24132375)
Supplement: Supplementary file 1 [file molecules-24-02375-s001.pdf]

Conserved G-quadruplexes regulate the Immediate Early Promoters of Human *Alphaherpesviruses*

Ilaria Frasson, Matteo Nadai and Sara N. Richter*

Department of Molecular Medicine, University of Padua, via A. Gabelli 63, 35121 Padua, Italy

Figure S1

A) CD thermal unfolding spectra, B) CD thermal unfolding fitting, and C) TDS of alphaherpesvirus IE promoter G4 sequences

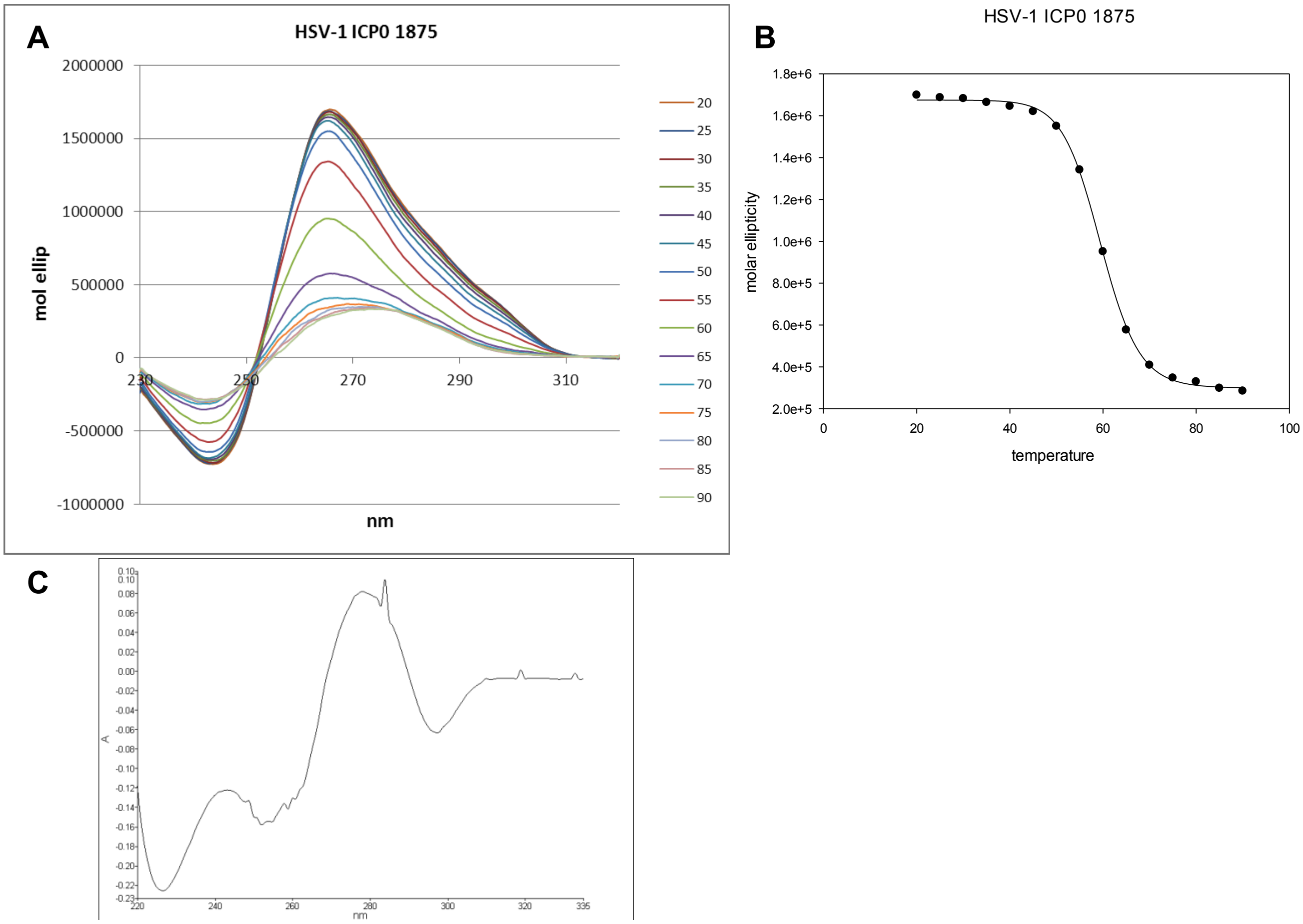

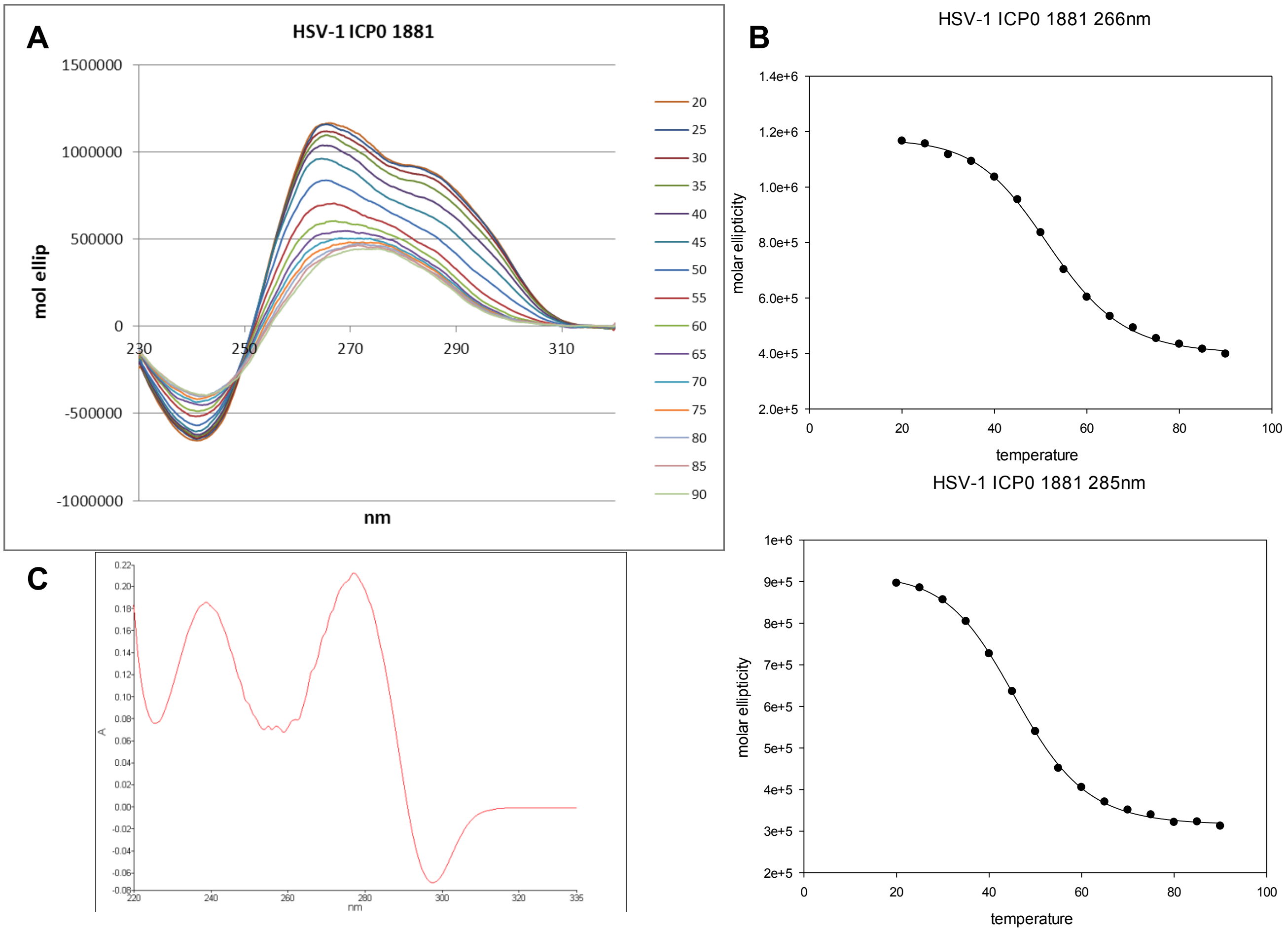
A
HSV-1 ICP0 1881
1500000
1000000
500000
0
-500000
-1000000
mol ellip
230
250
270
290
310
nm
20
25
30
35
40
45
50
55
60
65
70
75
80
85
90
B
HSV-1 ICP0 1881 266nm
1.4e+6
1.2e+6
1.0e+6
8.0e+5
6.0e+5
4.0e+5
2.0e+5
molar ellipticity
0
20
40
60
80
100
temperature
HSV-1 ICP0 1881 285nm
1e+6
9e+5
8e+5
7e+5
6e+5
5e+5
4e+5
3e+5
2e+5
molar ellipticity
0
20
40
60
80
100
temperature
C

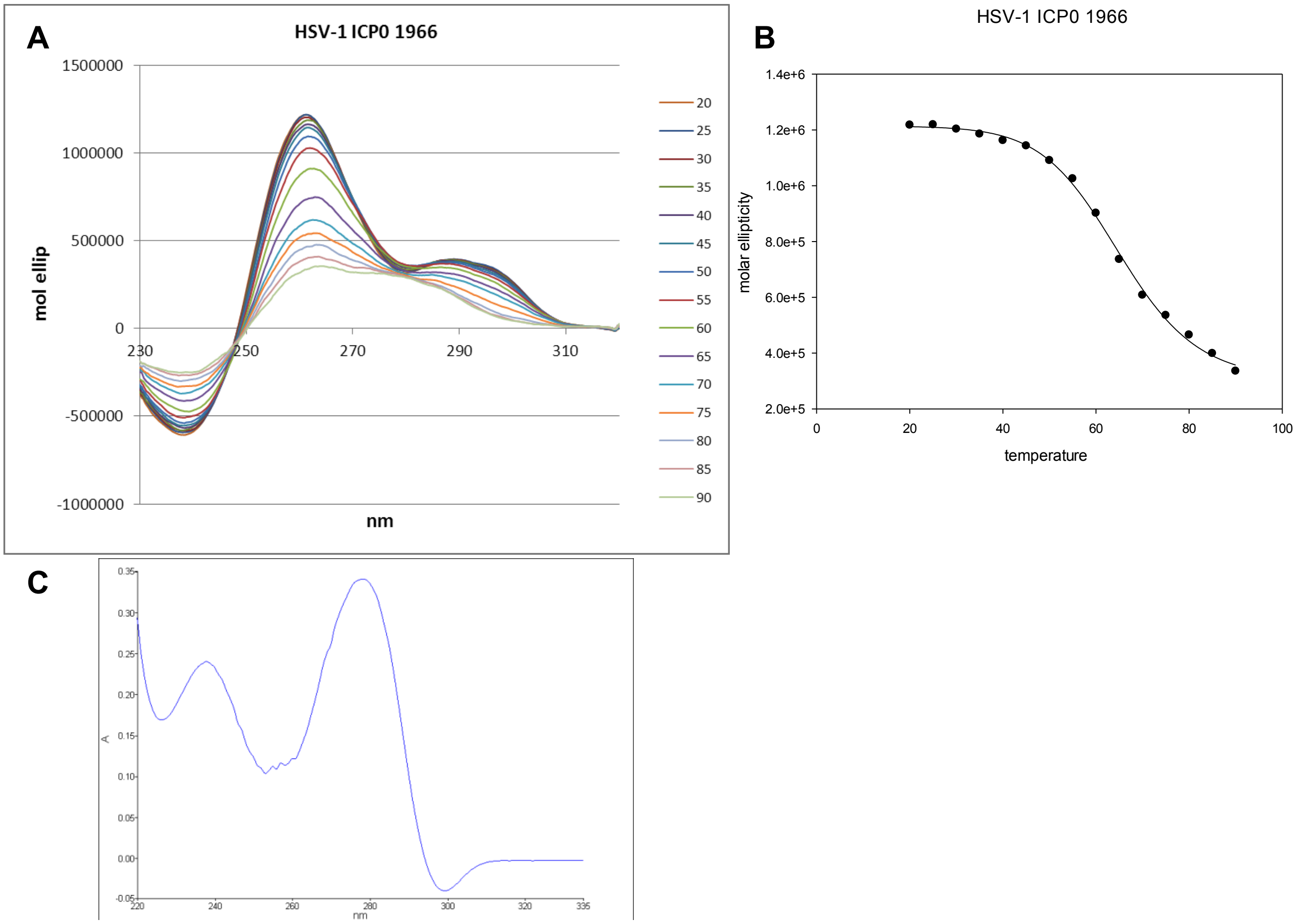
A
HSV-1 ICP0 1966
1500000
1000000
500000
0
-500000
-1000000
mol ellip
230
250
270
290
310
nm
20
25
30
35
40
45
50
55
60
65
70
75
80
85
90
B
HSV-1 ICP0 1966
1.4e+6
1.2e+6
1.0e+6
8.0e+5
6.0e+5
4.0e+5
2.0e+5
molar ellipticity
0
20
40
60
80
100
temperature
C

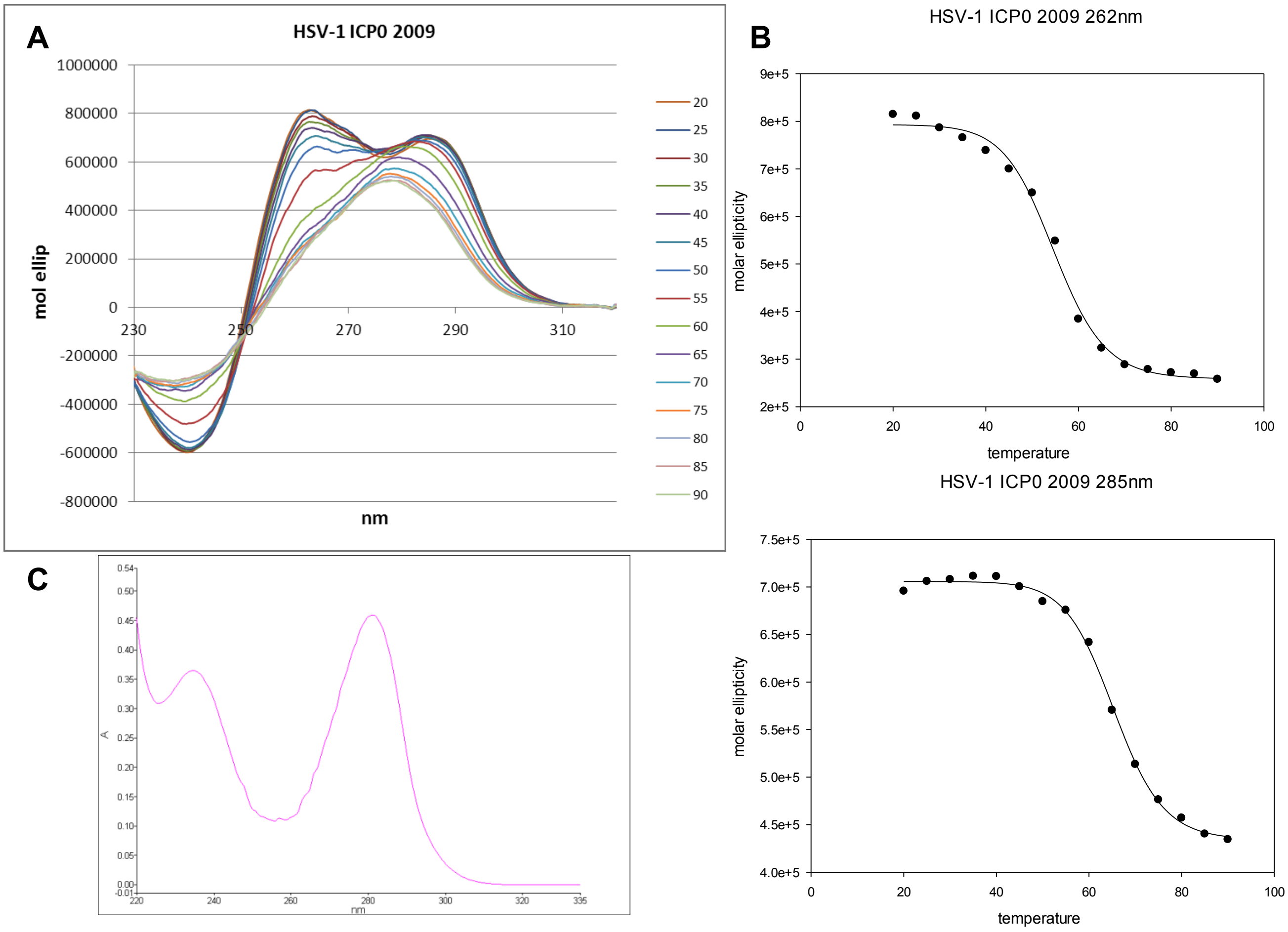
A
HSV-1 ICP0 2009
mol ellip
nm
1000000
800000
600000
400000
200000
0
-200000
-400000
-600000
-800000
230
250
270
290
310
20
25
30
35
40
45
50
55
60
65
70
75
80
85
90
B
HSV-1 ICP0 2009 262nm
molar ellipticity
temperature
9e+5
8e+5
7e+5
6e+5
5e+5
4e+5
3e+5
2e+5
0
20
40
60
80
100
HSV-1 ICP0 2009 285nm
molar ellipticity
temperature
7.5e+5
7.0e+5
6.5e+5
6.0e+5
5.5e+5
5.0e+5
4.5e+5
4.0e+5
0
20
40
60
80
100
C

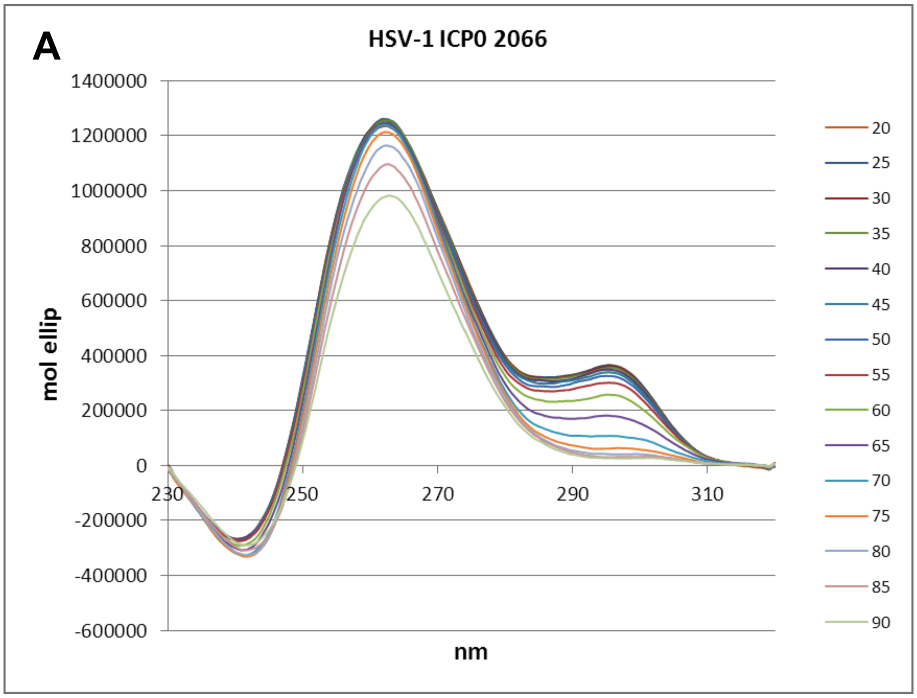
A
HSV-1 ICP0 2066
mol ellip
nm
1400000
1200000
1000000
800000
600000
400000
200000
0
-200000
-400000
-600000
230
250
270
290
310
20
25
30
35
40
45
50
55
60
65
70
75
80
85
90

B

T_m >90

C

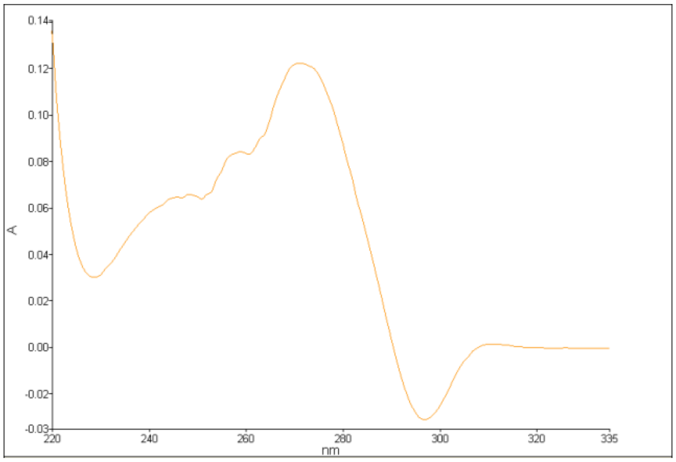
A
nm
220
240
260
280
300
320
335

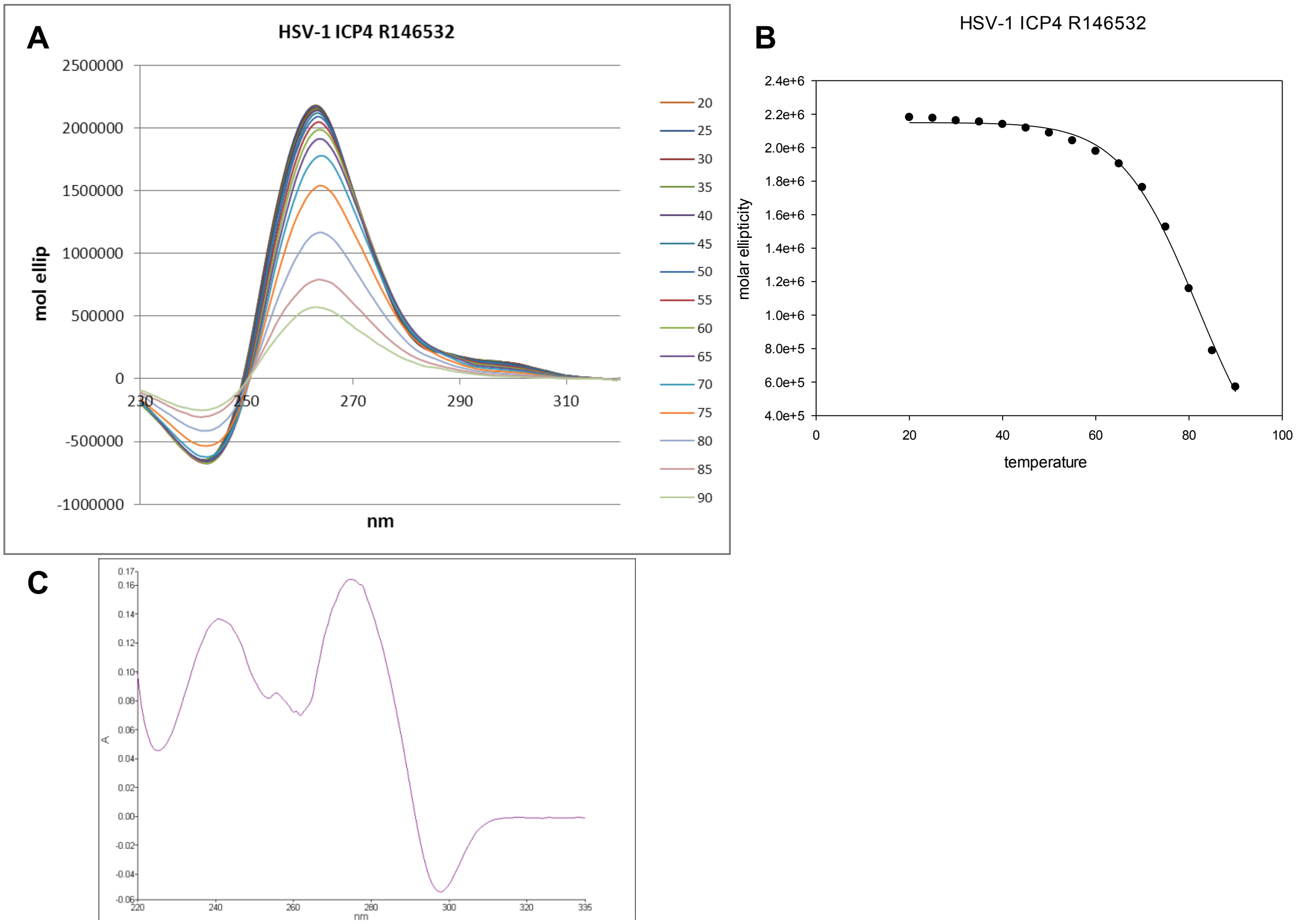

A
HSV-1 ICP4 R146532
2500000
2000000
1500000
1000000
500000
0
-500000
-1000000
mol ellip
230
250
270
290
310
nm
20
25
30
35
40
45
50
55
60
65
70
75
80
85
90
B
HSV-1 ICP4 R146532
2.4e+6
2.2e+6
2.0e+6
1.8e+6
1.6e+6
1.4e+6
1.2e+6
1.0e+6
8.0e+5
6.0e+5
4.0e+5
molar ellipticity
0
20
40
60
80
100
temperature
C
0.17
0.16
0.14
0.12
0.10
0.08
0.06
0.04
0.02
0.00
-0.02
-0.04
-0.06
A
220
240
260
280
300
320
335
nm

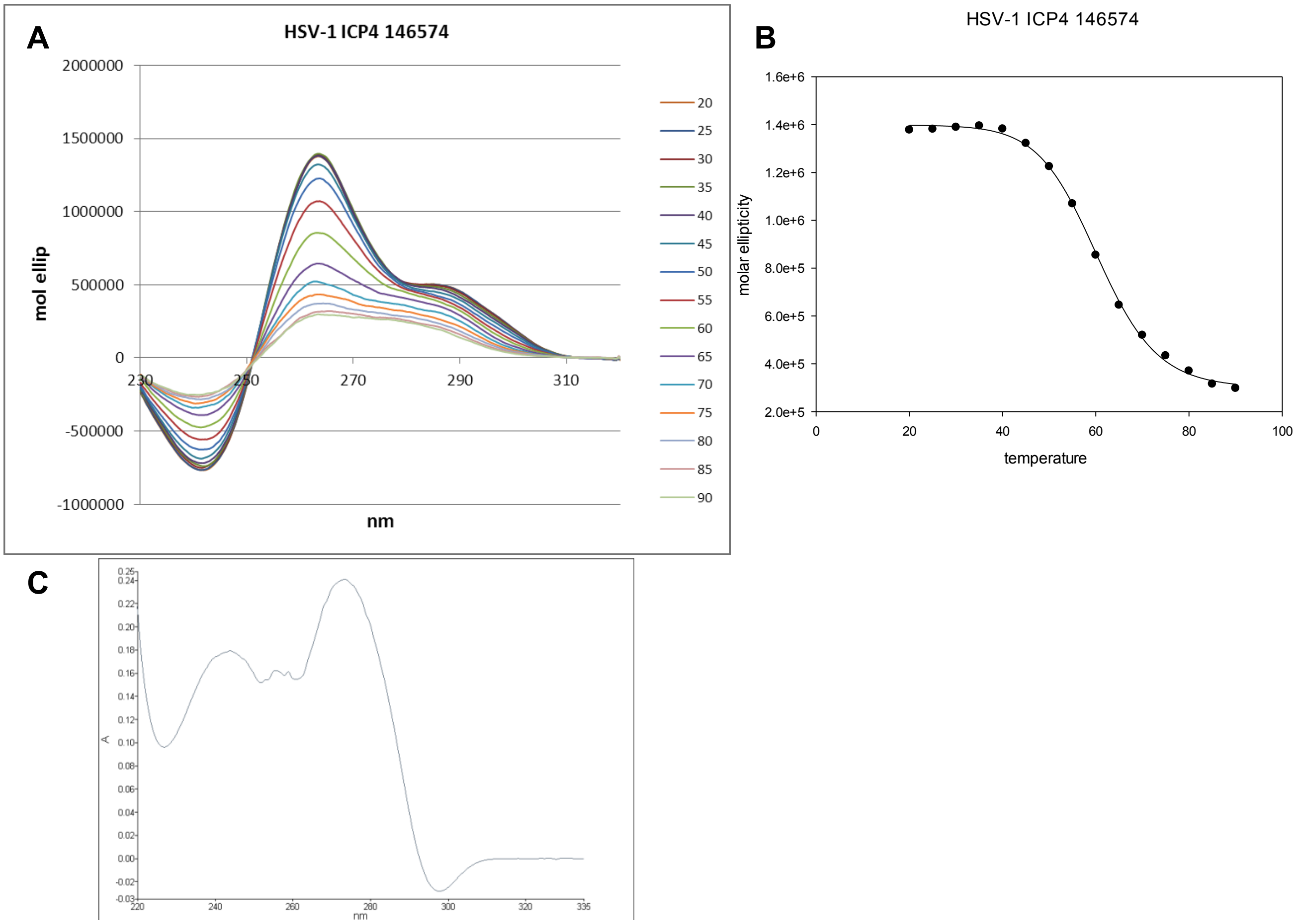
A
HSV-1 ICP4 146574
mol ellip
nm
2000000
1500000
1000000
500000
0
-500000
-1000000
230
250
270
290
310
20
25
30
35
40
45
50
55
60
65
70
75
80
85
90
B
HSV-1 ICP4 146574
molar ellipticity
temperature
1.6e+6
1.4e+6
1.2e+6
1.0e+6
8.0e+5
6.0e+5
4.0e+5
2.0e+5
0
20
40
60
80
100
C
A
nm
220
240
260
280
300
320
335

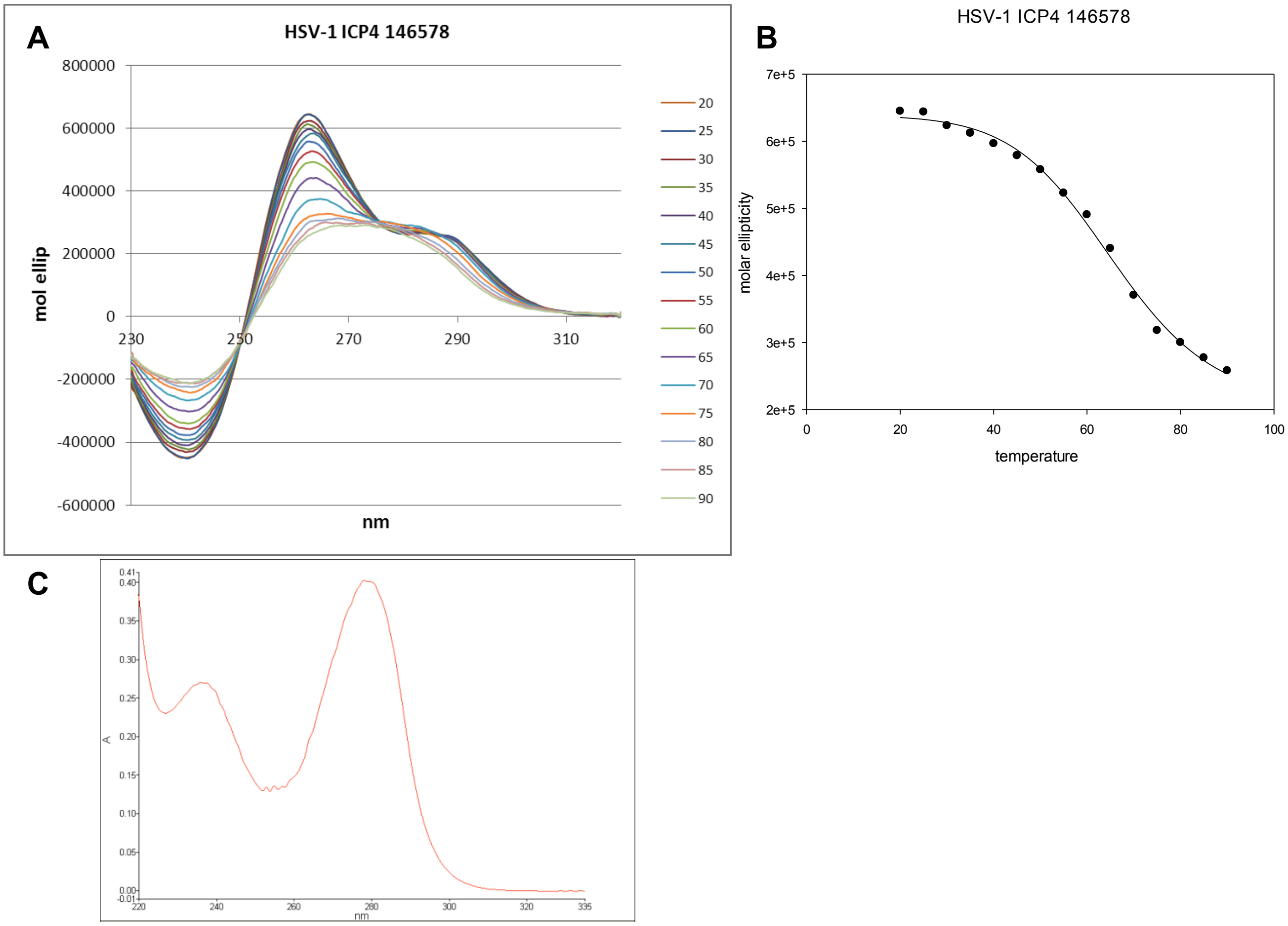
A
HSV-1 ICP4 146578
800000
600000
400000
200000
0
-200000
-400000
-600000
mol ellip
230
250
270
290
310
nm
20
25
30
35
40
45
50
55
60
65
70
75
80
85
90
B
HSV-1 ICP4 146578
7e+5
6e+5
5e+5
4e+5
3e+5
2e+5
molar ellipticity
0
20
40
60
80
100
temperature
C
0.41
0.40
0.35
0.30
0.25
0.20
0.15
0.10
0.05
0.00
-0.01
A
220
240
260
280
300
320
335
nm

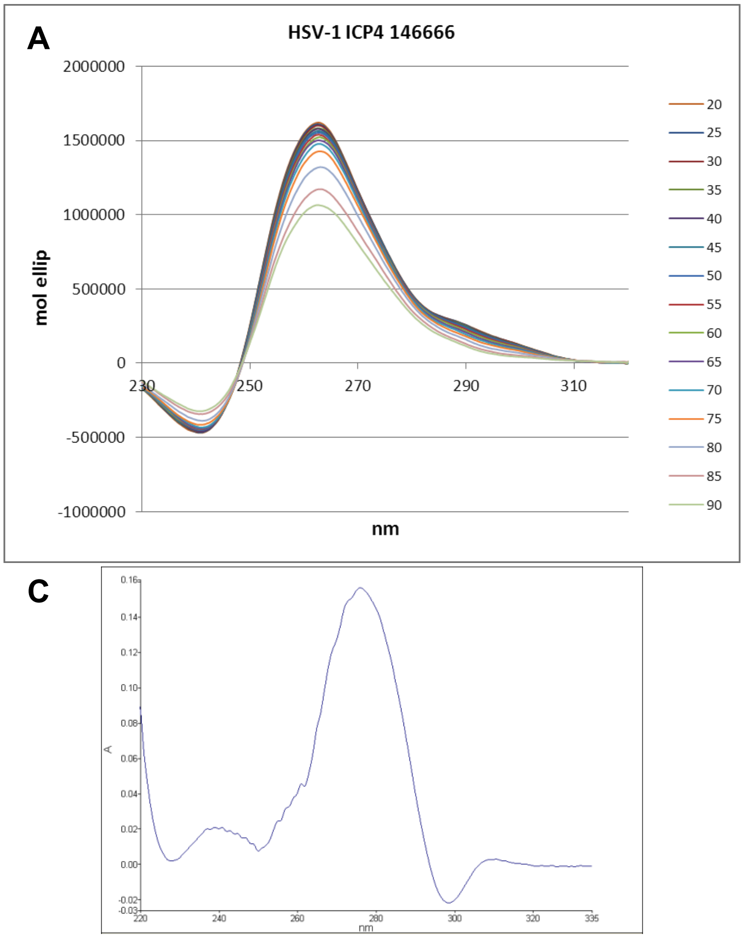
A
HSV-1 ICP4 146666
mol ellip
nm
2000000
1500000
1000000
500000
0
-500000
-1000000
230
250
270
290
310
20
25
30
35
40
45
50
55
60
65
70
75
80
85
90
C
A
nm
0.16
0.14
0.12
0.10
0.08
0.06
0.04
0.02
0.00
-0.02
-0.03
220
240
260
280
300
320
335

B

T_m >90

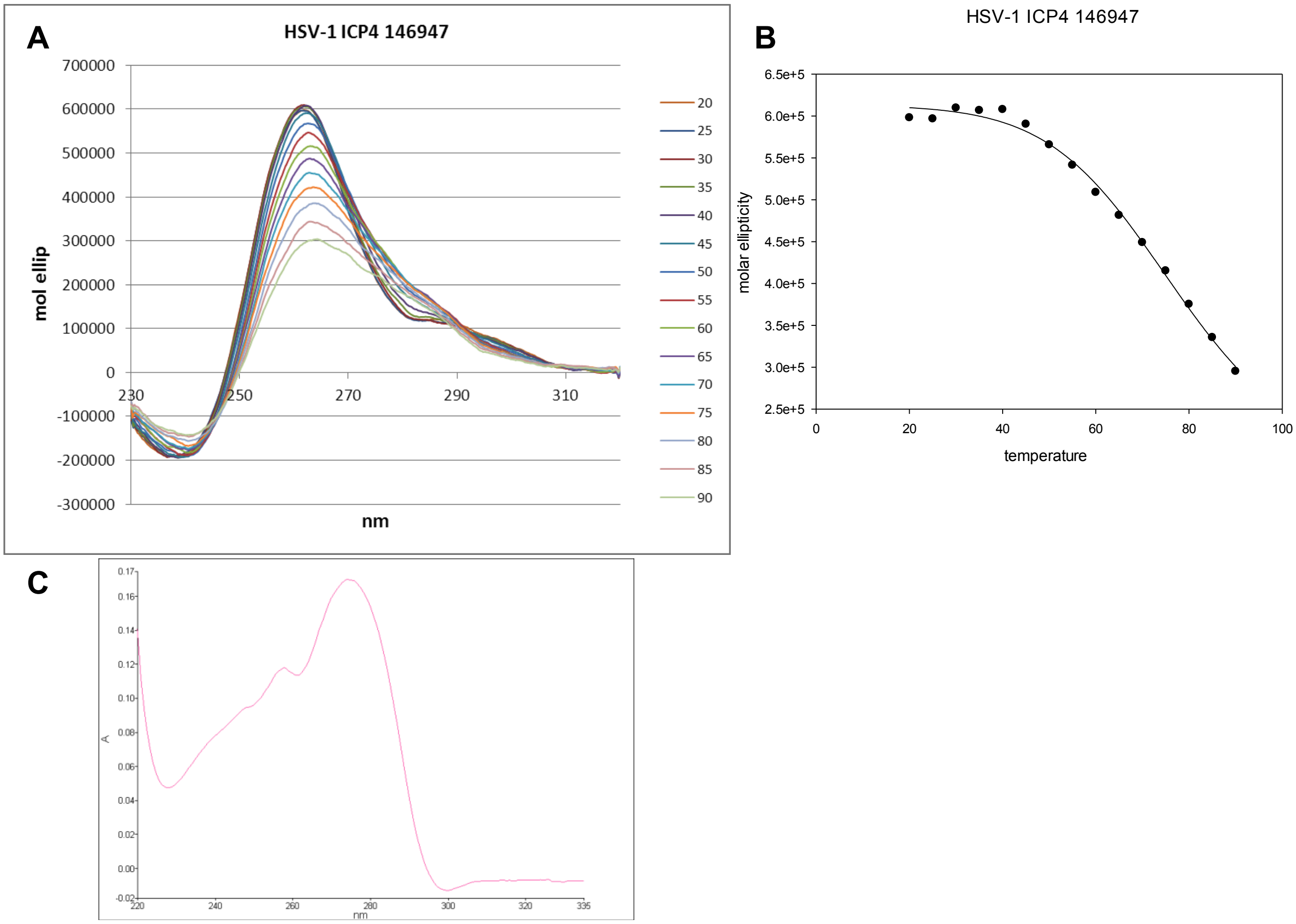
A
HSV-1 ICP4 146947
700000
600000
500000
400000
300000
200000
100000
0
-100000
-200000
-300000
mol ellip
230
250
270
290
310
nm
20
25
30
35
40
45
50
55
60
65
70
75
80
85
90
B
HSV-1 ICP4 146947
6.5e+5
6.0e+5
5.5e+5
5.0e+5
4.5e+5
4.0e+5
3.5e+5
3.0e+5
2.5e+5
molar ellipticity
0
20
40
60
80
100
temperature
C

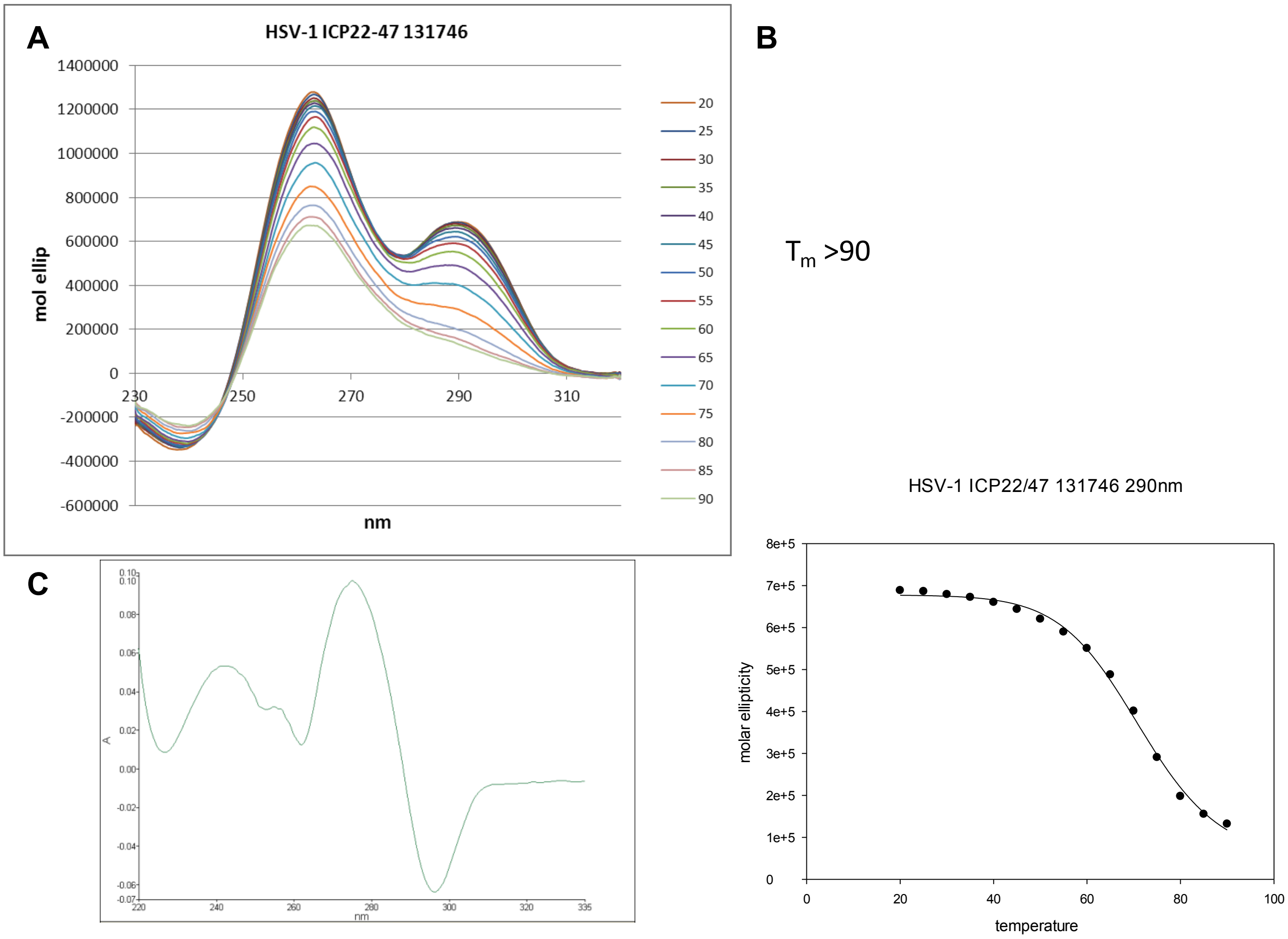

A
HSV-1 ICP22-47 131746
mol ellip
nm
20
25
30
35
40
45
50
55
60
65
70
75
80
85
90
B
T_m >90
HSV-1 ICP22/47 131746 290nm
molar ellipticity
temperature
C
A
nm

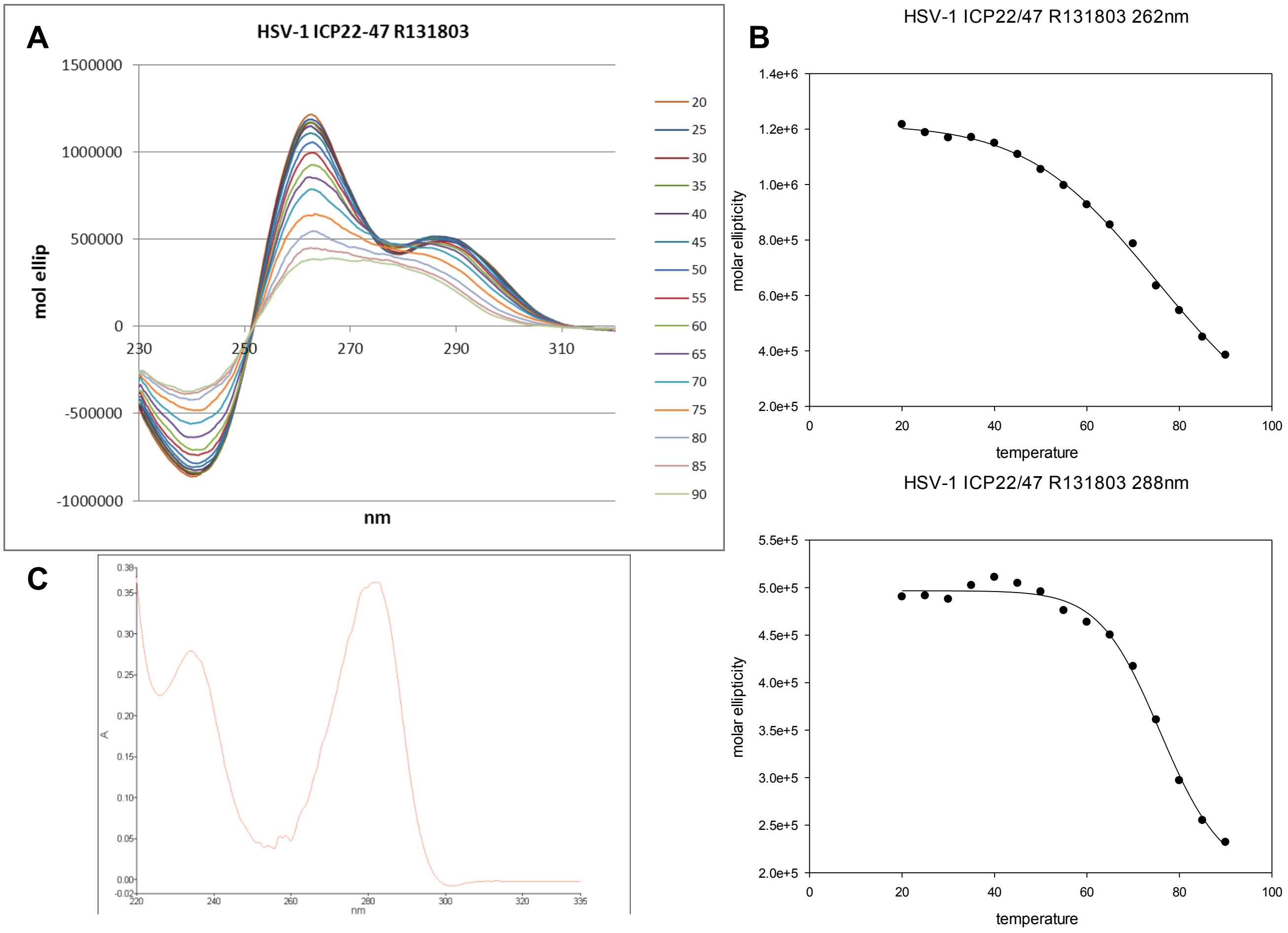

A
HSV-1 ICP22-47 R131803
1500000
1000000
500000
0
-500000
-1000000
mol ellip
230
250
270
290
310
nm
20
25
30
35
40
45
50
55
60
65
70
75
80
85
90
B
HSV-1 ICP22/47 R131803 262nm
1.4e+6
1.2e+6
1.0e+6
8.0e+5
6.0e+5
4.0e+5
2.0e+5
molar ellipticity
0
20
40
60
80
100
temperature
HSV-1 ICP22/47 R131803 288nm
5.5e+5
5.0e+5
4.5e+5
4.0e+5
3.5e+5
3.0e+5
2.5e+5
2.0e+5
molar ellipticity
0
20
40
60
80
100
temperature
C

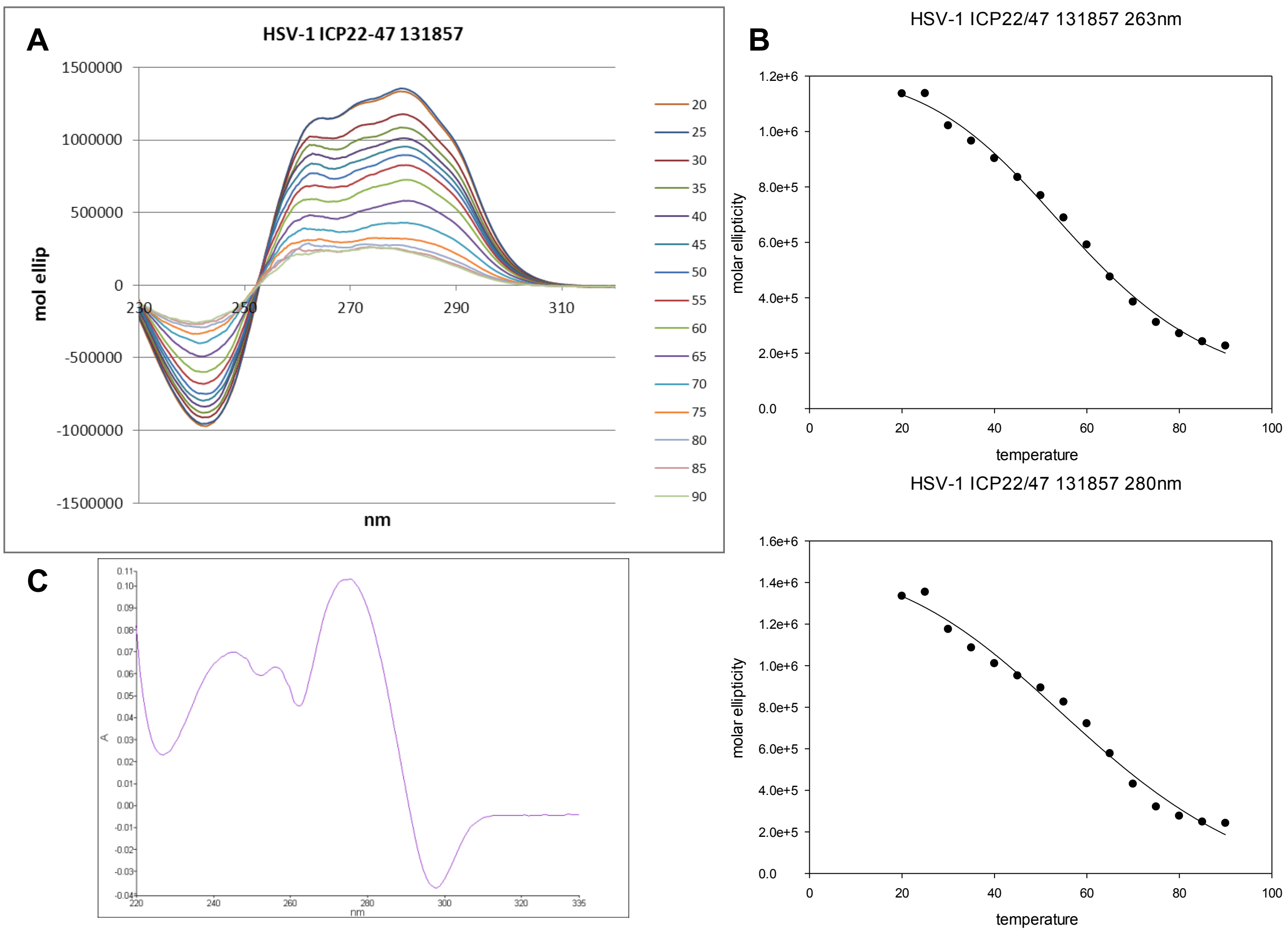
A
HSV-1 ICP22-47 131857
1500000
1000000
500000
0
-500000
-1000000
-1500000
mol ellip
230
250
270
290
310
nm
20
25
30
35
40
45
50
55
60
65
70
75
80
85
90
B
HSV-1 ICP22/47 131857 263nm
1.2e+6
1.0e+6
8.0e+5
6.0e+5
4.0e+5
2.0e+5
0.0
molar ellipticity
0
20
40
60
80
100
temperature
HSV-1 ICP22/47 131857 280nm
1.6e+6
1.4e+6
1.2e+6
1.0e+6
8.0e+5
6.0e+5
4.0e+5
2.0e+5
0.0
molar ellipticity
0
20
40
60
80
100
temperature
C
A
nm
220
240
260
280
300
320
335

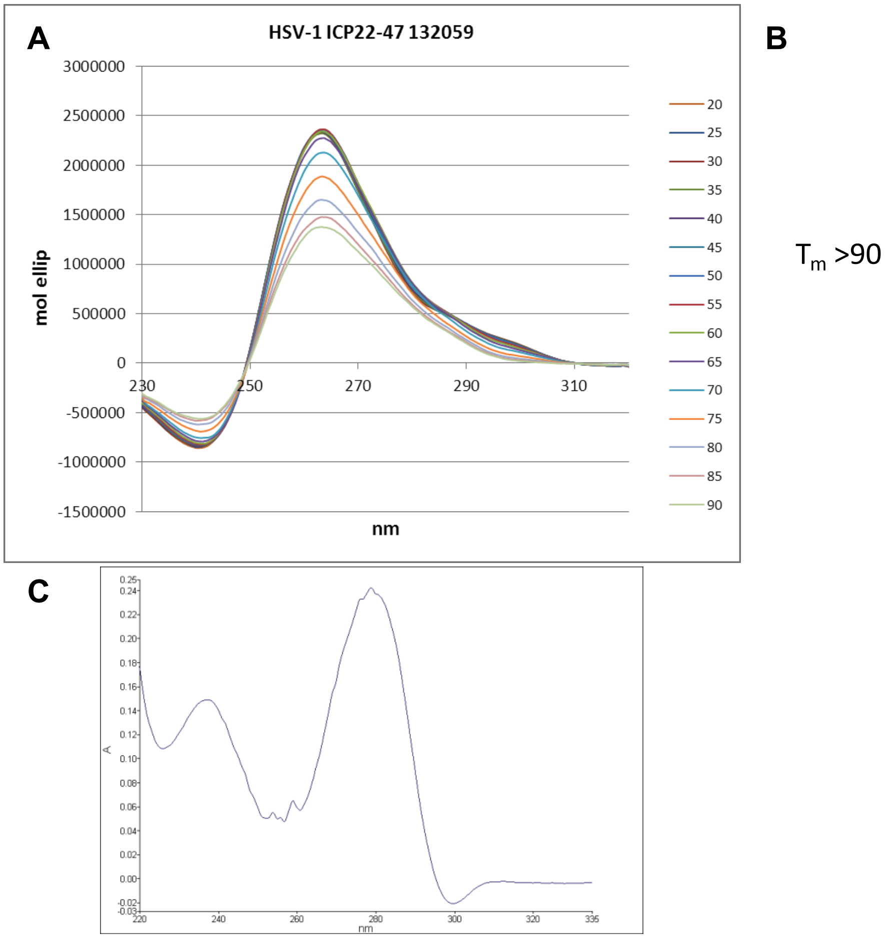

A
HSV-1 ICP22-47 132059
mol ellip
nm
20
25
30
35
40
45
50
55
60
65
70
75
80
85
90
B
T_m >90
C
A
nm

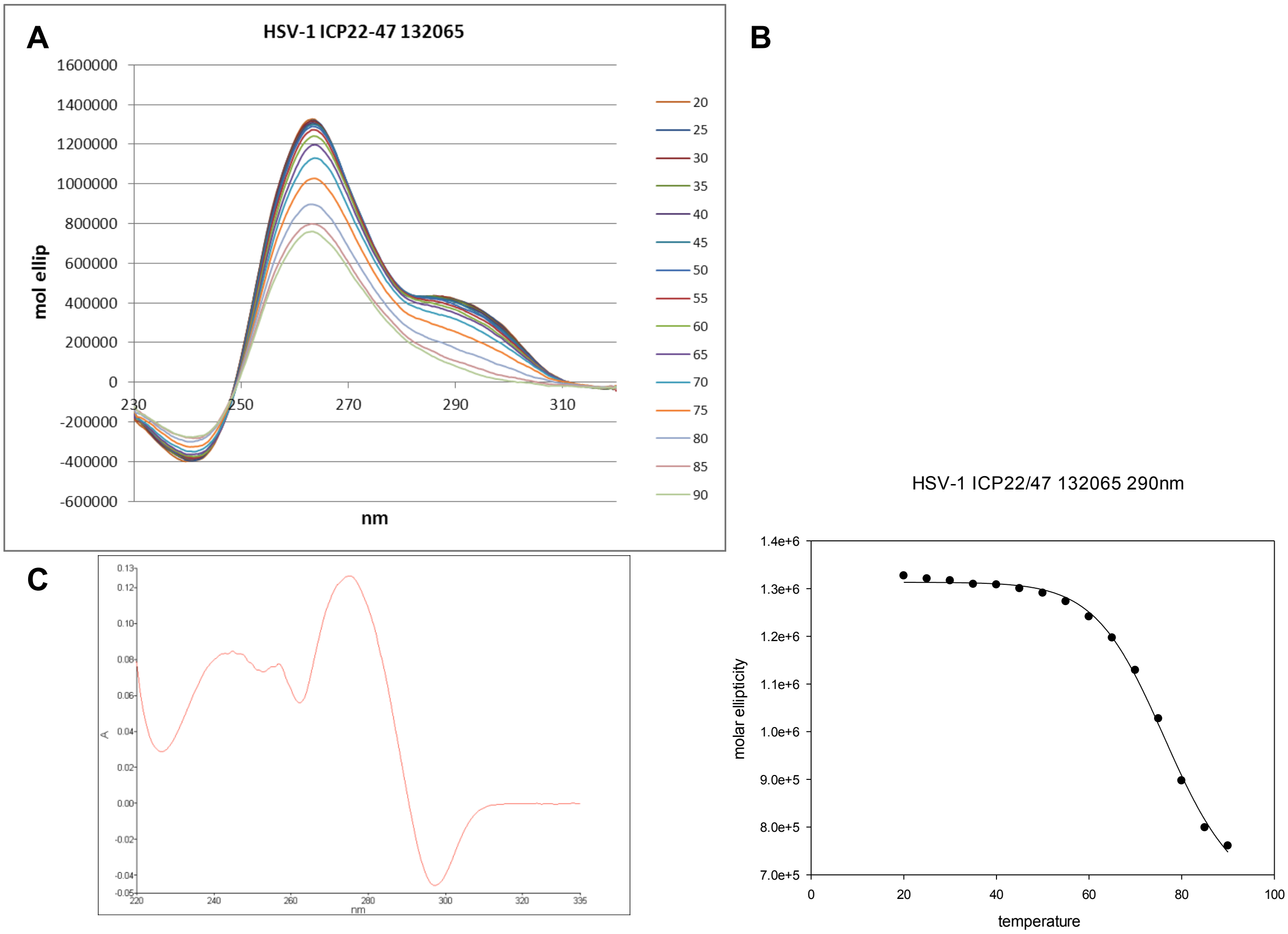

A
HSV-1 ICP22-47 132065
mol ellip
1600000
1400000
1200000
1000000
800000
600000
400000
200000
0
-200000
-400000
-600000
230
250
270
290
310
nm
20
25
30
35
40
45
50
55
60
65
70
75
80
85
90
B
HSV-1 ICP22/47 132065 290nm
molar ellipticity
1.4e+6
1.3e+6
1.2e+6
1.1e+6
1.0e+6
9.0e+5
8.0e+5
7.0e+5
0
20
40
60
80
100
temperature
C
A
nm

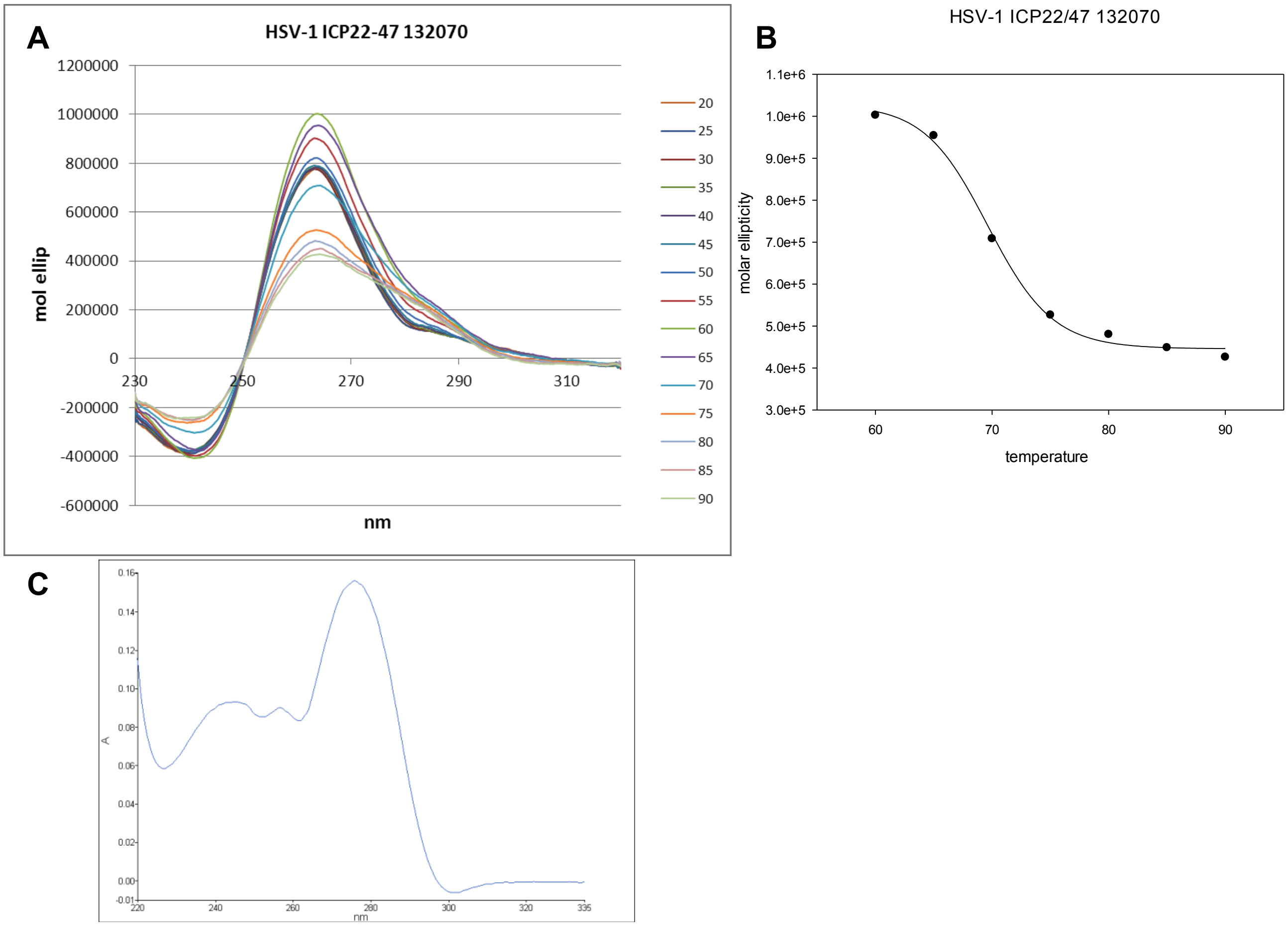
A
HSV-1 ICP22-47 132070
1200000
1000000
800000
600000
400000
200000
0
-200000
-400000
-600000
mol ellip
230
250
270
290
310
nm
20
25
30
35
40
45
50
55
60
65
70
75
80
85
90
B
HSV-1 ICP22/47 132070
1.1e+6
1.0e+6
9.0e+5
8.0e+5
7.0e+5
6.0e+5
5.0e+5
4.0e+5
3.0e+5
molar ellipticity
60
70
80
90
temperature
C
0.16
0.14
0.12
0.10
0.08
0.06
0.04
0.02
0.00
-0.01
A
220
240
260
280
300
320
335
nm

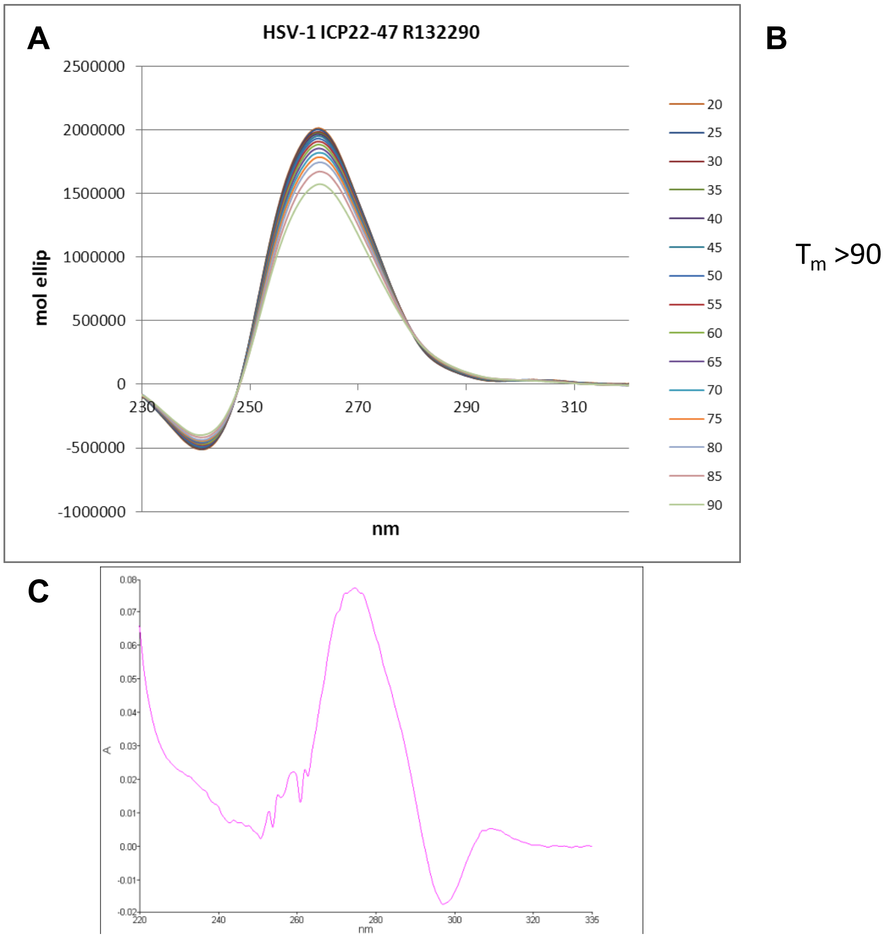
A
HSV-1 ICP22-47 R132290
2500000
2000000
1500000
1000000
500000
0
-500000
-1000000
mol ellip
230
250
270
290
310
nm
20
25
30
35
40
45
50
55
60
65
70
75
80
85
90
B
T_m >90
C
0.08
0.07
0.06
0.05
0.04
0.03
0.02
0.01
0.00
-0.01
-0.02
A
220
240
260
280
300
320
335
nm

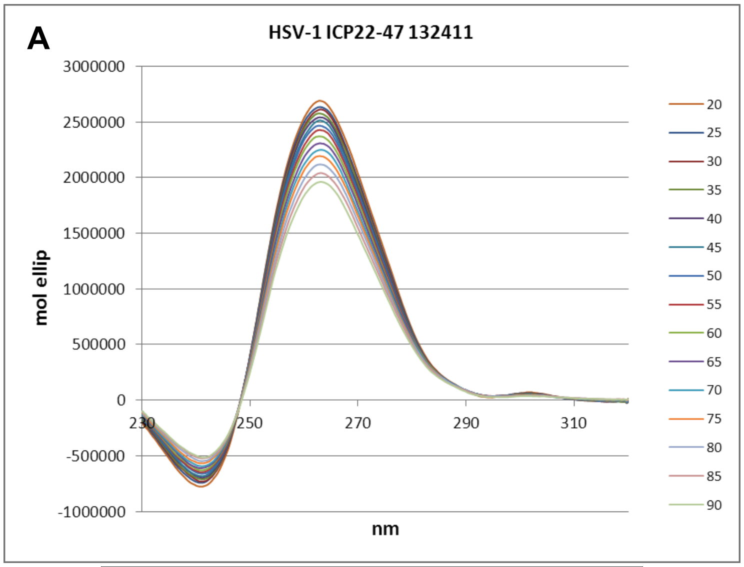
A
HSV-1 ICP22-47 132411
mol ellip
3000000
2500000
2000000
1500000
1000000
500000
0
-500000
-1000000
230
250
270
290
310
nm
20
25
30
35
40
45
50
55
60
65
70
75
80
85
90

B

T_m >90

C

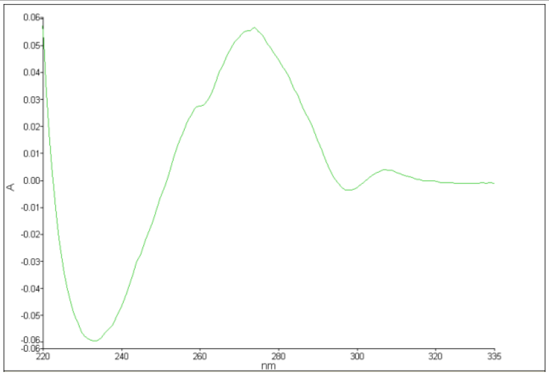
0.06
0.05
0.04
0.03
0.02
0.01
0.00
-0.01
-0.02
-0.03
-0.04
-0.05
-0.06
A
220
240
260
280
300
320
335
nm

A

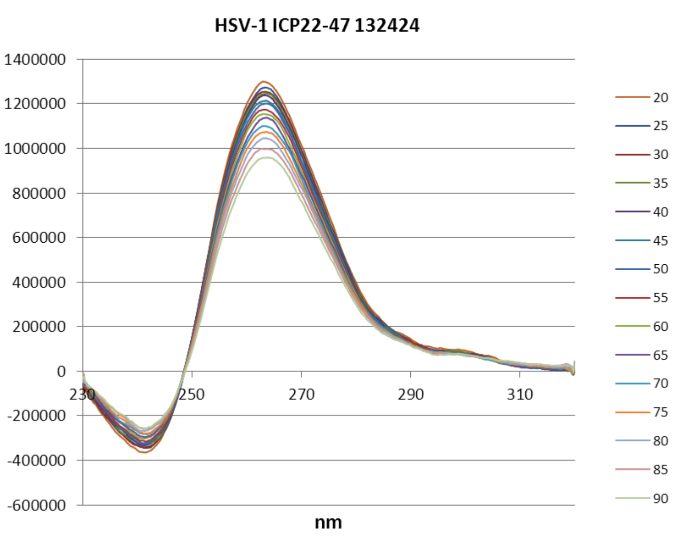
HSV-1 ICP22-47 132424
1400000
1200000
1000000
800000
600000
400000
200000
0
-200000
-400000
-600000
mol ellip
230
250
270
290
310
nm
20
25
30
35
40
45
50
55
60
65
70
75
80
85
90

B

T_m >90

C

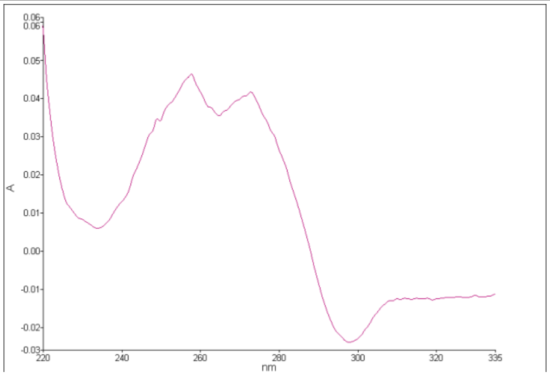
0.06
0.05
0.04
0.03
0.02
0.01
0.00
-0.01
-0.02
-0.03
A
220
240
260
280
300
320
335
nm

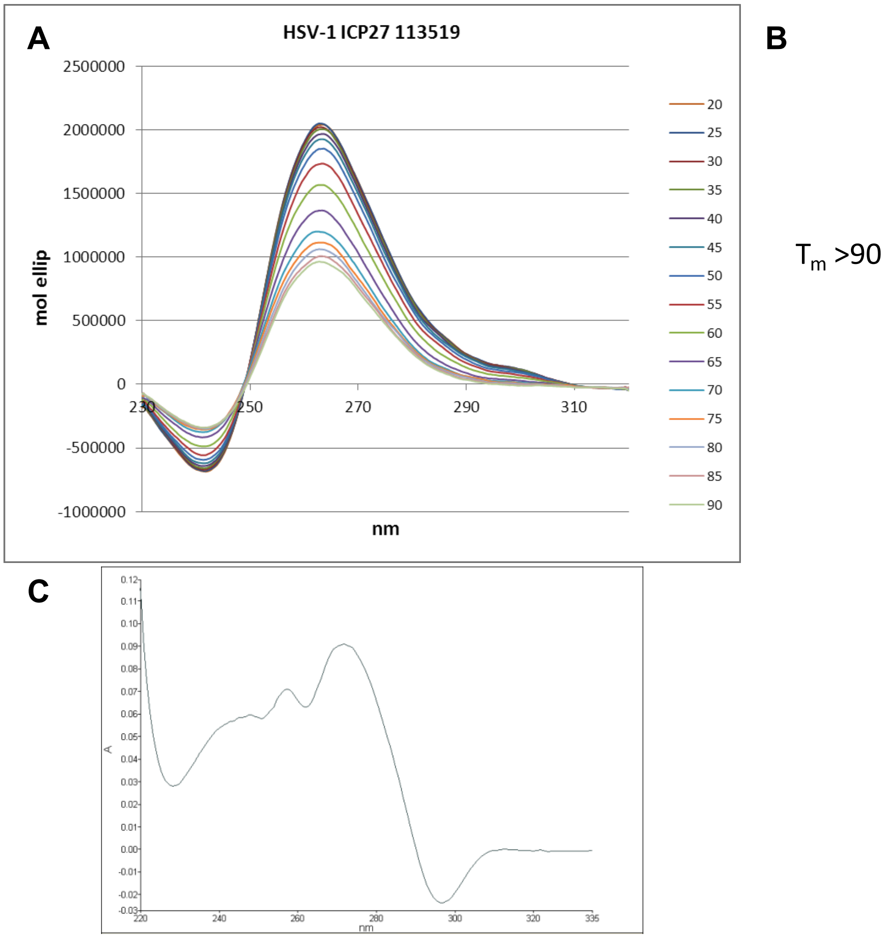
A
HSV-1 ICP27 113519
mol ellip
nm
2500000
2000000
1500000
1000000
500000
0
-500000
-1000000
230
250
270
290
310
20
25
30
35
40
45
50
55
60
65
70
75
80
85
90
B
T_m >90
C
A
nm
220
240
260
280
300
320
335

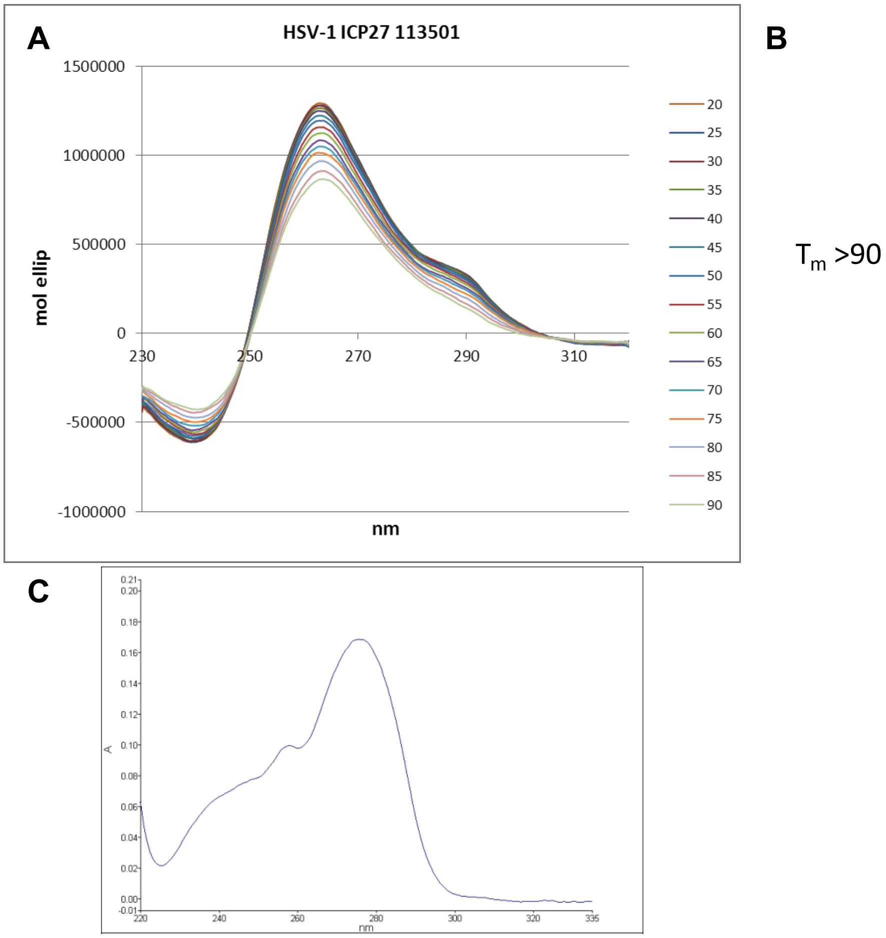
A
HSV-1 ICP27 113501
1500000
1000000
500000
0
-500000
-1000000
mol ellip
230
250
270
290
310
nm
20
25
30
35
40
45
50
55
60
65
70
75
80
85
90
B
T_m >90
C
0.21
0.20
0.18
0.16
0.14
0.12
0.10
0.08
0.06
0.04
0.02
0.00
-0.01
A
220
240
260
280
300
320
335
nm

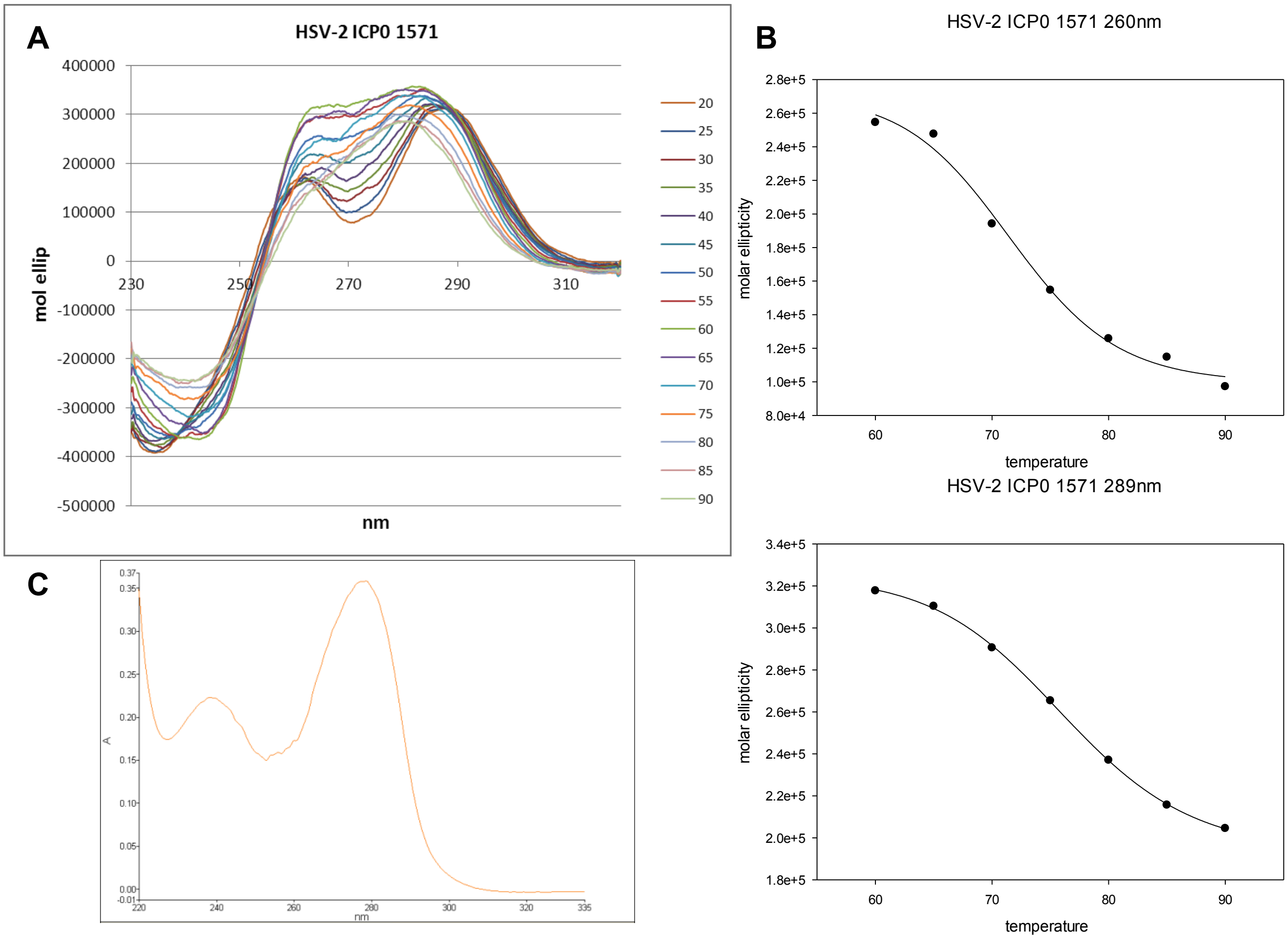
A
HSV-2 ICP0 1571
400000
300000
200000
100000
0
-100000
-200000
-300000
-400000
-500000
mol ellip
230
250
270
290
310
nm
20
25
30
35
40
45
50
55
60
65
70
75
80
85
90
B
HSV-2 ICP0 1571 260nm
2.8e+5
2.6e+5
2.4e+5
2.2e+5
2.0e+5
1.8e+5
1.6e+5
1.4e+5
1.2e+5
1.0e+5
8.0e+4
molar ellipticity
60
70
80
90
temperature
HSV-2 ICP0 1571 289nm
3.4e+5
3.2e+5
3.0e+5
2.8e+5
2.6e+5
2.4e+5
2.2e+5
2.0e+5
1.8e+5
molar ellipticity
60
70
80
90
temperature
C
0.37
0.35
0.30
0.25
0.20
0.15
0.10
0.05
0.00
-0.01
A
220
240
260
280
300
320
335
nm

A

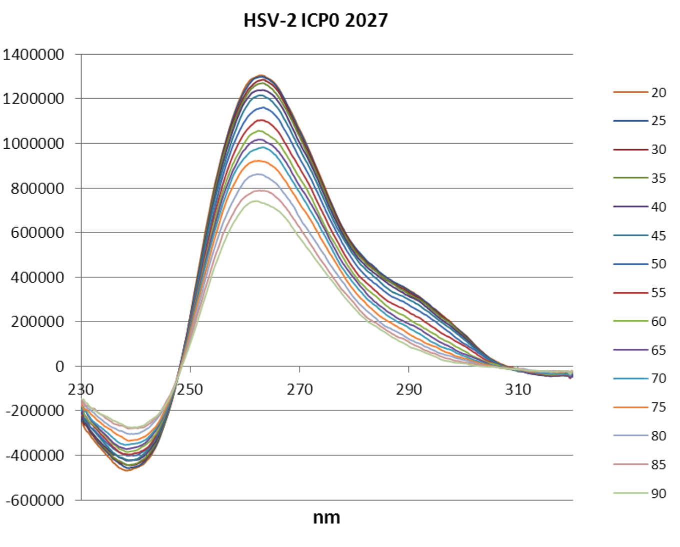
HSV-2 ICP0 2027
1400000
1200000
1000000
800000
600000
400000
200000
0
-200000
-400000
-600000
mol ellip
230
250
270
290
310
nm
20
25
30
35
40
45
50
55
60
65
70
75
80
85
90

B

T_m >90

C

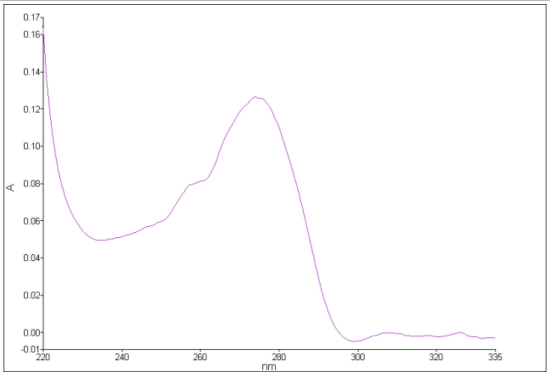
0.17
0.16
0.14
0.12
0.10
0.08
0.06
0.04
0.02
0.00
-0.01
A
220
240
260
280
300
320
335
nm

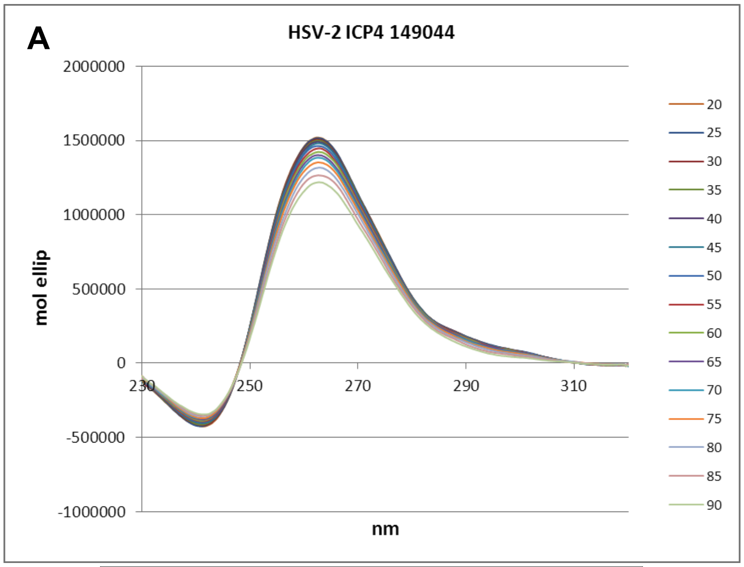
A
HSV-2 ICP4 149044
2000000
1500000
1000000
500000
0
-500000
-1000000
mol ellip
230
250
270
290
310
nm
20
25
30
35
40
45
50
55
60
65
70
75
80
85
90

B

T_m >90

C

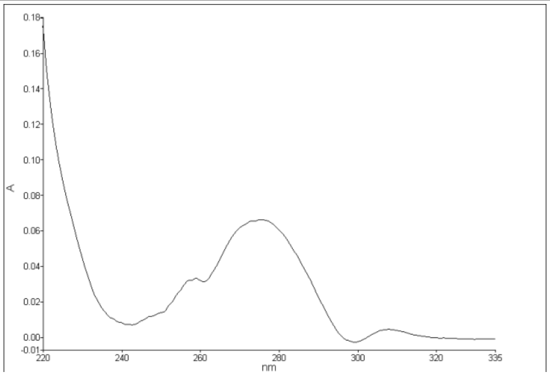
0.18
0.16
0.14
0.12
0.10
0.08
0.06
0.04
0.02
0.00
-0.01
A
220
240
260
280
300
320
335
nm

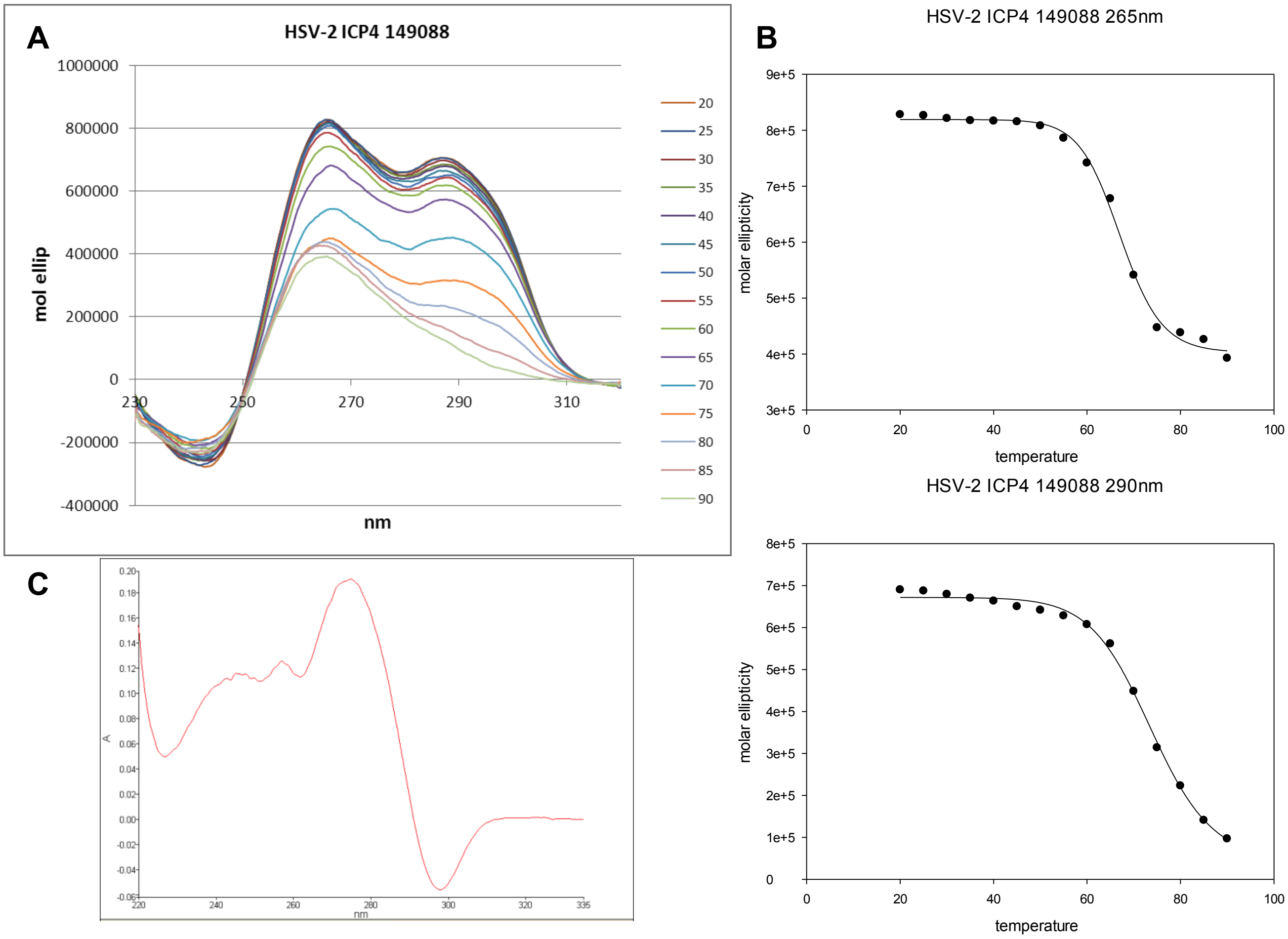
A
HSV-2 ICP4 149088
mol ellip
nm
1000000
800000
600000
400000
200000
0
-200000
-400000
230
250
270
290
310
20
25
30
35
40
45
50
55
60
65
70
75
80
85
90
B
HSV-2 ICP4 149088 265nm
molar ellipticity
temperature
9e+5
8e+5
7e+5
6e+5
5e+5
4e+5
3e+5
0
20
40
60
80
100
HSV-2 ICP4 149088 290nm
molar ellipticity
temperature
8e+5
7e+5
6e+5
5e+5
4e+5
3e+5
2e+5
1e+5
0
C

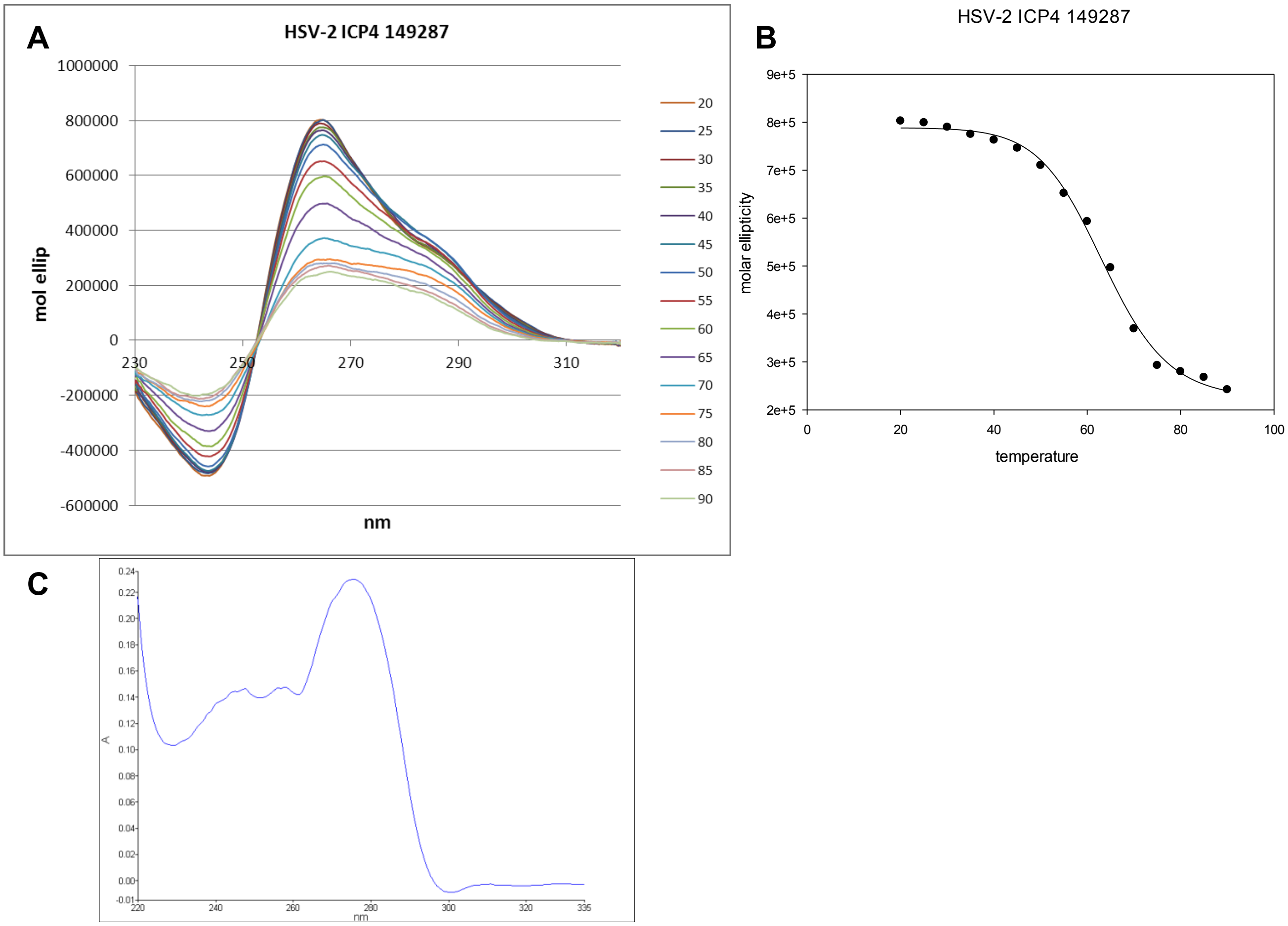
A
HSV-2 ICP4 149287
1000000
800000
600000
400000
200000
0
-200000
-400000
-600000
mol ellip
230
250
270
290
310
nm
20
25
30
35
40
45
50
55
60
65
70
75
80
85
90
B
HSV-2 ICP4 149287
9e+5
8e+5
7e+5
6e+5
5e+5
4e+5
3e+5
2e+5
molar ellipticity
0
20
40
60
80
100
temperature
C
0.24
0.22
0.20
0.18
0.16
0.14
0.12
0.10
0.08
0.06
0.04
0.02
0.00
-0.01
A
220
240
260
280
300
320
335
nm

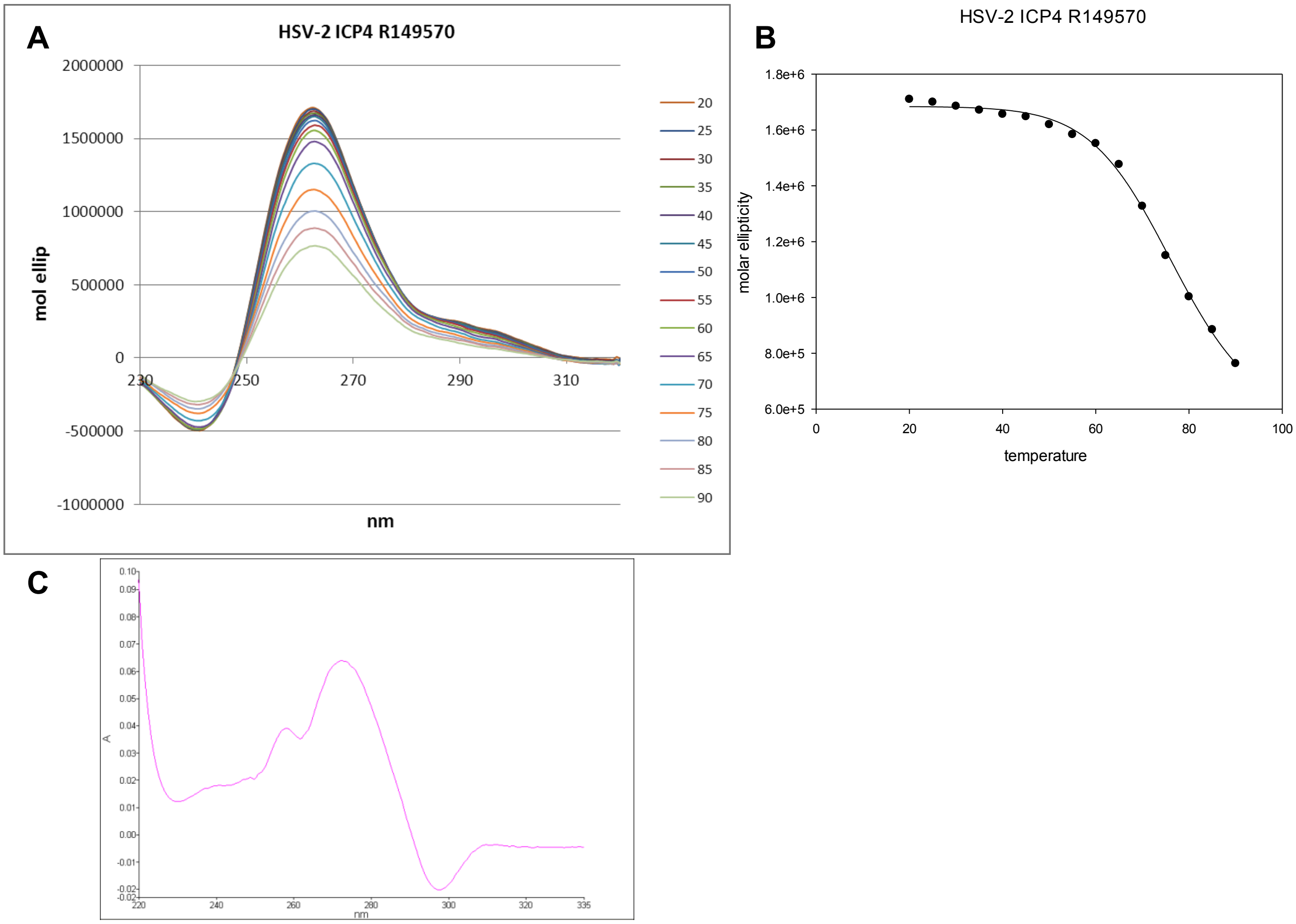

A
HSV-2 ICP4 R149570
mol ellip
2000000
1500000
1000000
500000
0
-500000
-1000000
230
250
270
290
310
nm
20
25
30
35
40
45
50
55
60
65
70
75
80
85
90
B
HSV-2 ICP4 R149570
molar ellipticity
1.8e+6
1.6e+6
1.4e+6
1.2e+6
1.0e+6
8.0e+5
6.0e+5
0
20
40
60
80
100
temperature
C

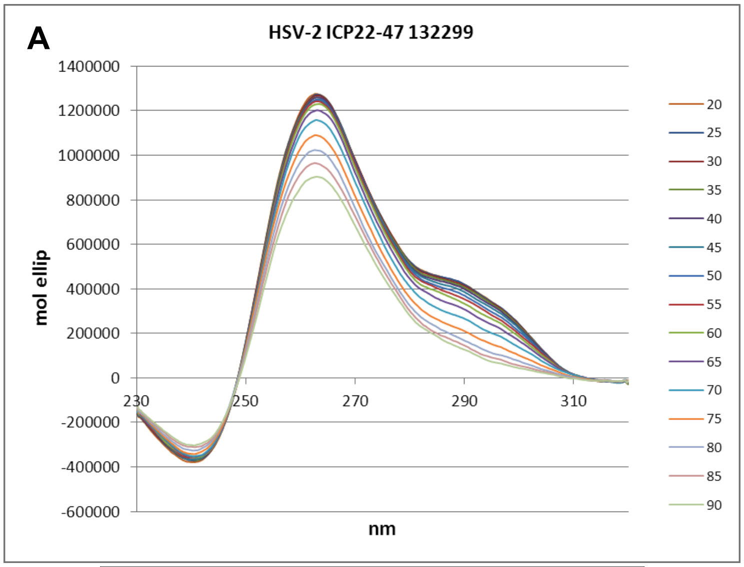
A
HSV-2 ICP22-47 132299
1400000
1200000
1000000
800000
600000
400000
200000
0
-200000
-400000
-600000
mol ellip
230
250
270
290
310
nm
20
25
30
35
40
45
50
55
60
65
70
75
80
85
90

B

T_m >90

C

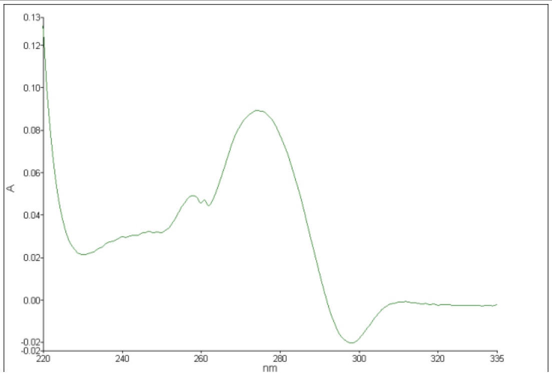
0.13
0.12
0.10
0.08
0.06
0.04
0.02
0.00
-0.02
A
220
240
260
280
300
320
335
nm

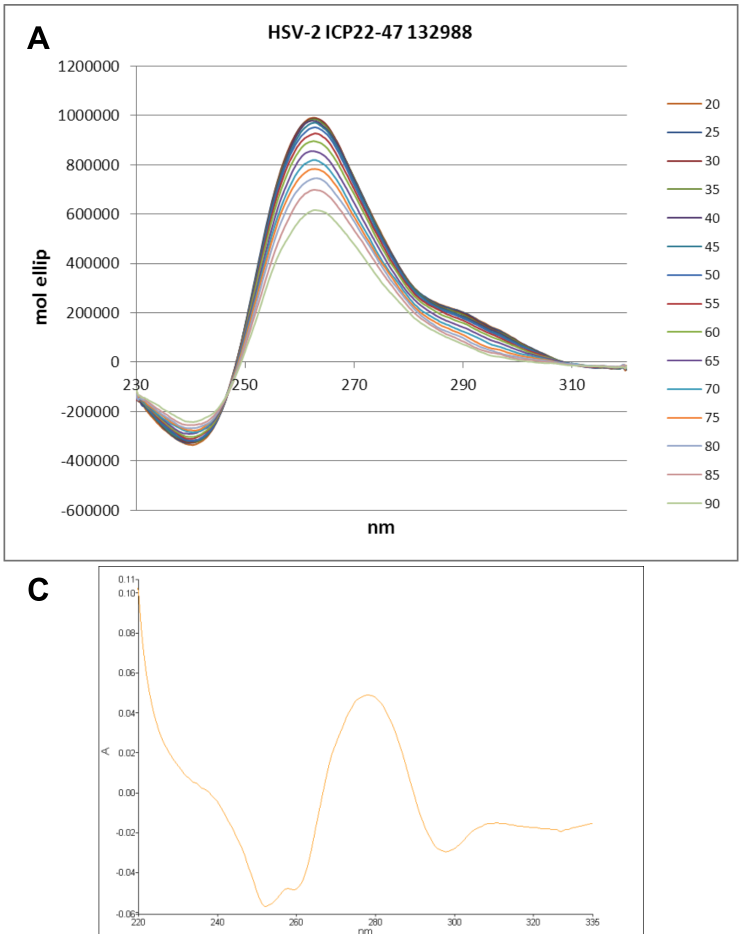
A
HSV-2 ICP22-47 132988
mol ellip
nm
1200000
1000000
800000
600000
400000
200000
0
-200000
-400000
-600000
230
250
270
290
310
20
25
30
35
40
45
50
55
60
65
70
75
80
85
90
C
A
nm
220
240
260
280
300
320
335

B

T_m >90

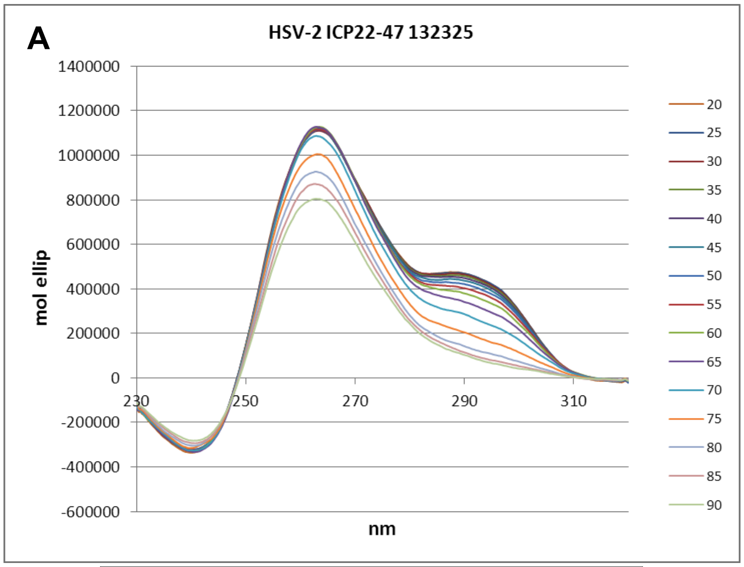
A
HSV-2 ICP22-47 132325
1400000
1200000
1000000
800000
600000
400000
200000
0
-200000
-400000
-600000
mol ellip
230
250
270
290
310
nm
20
25
30
35
40
45
50
55
60
65
70
75
80
85
90

B

T_m >90

C

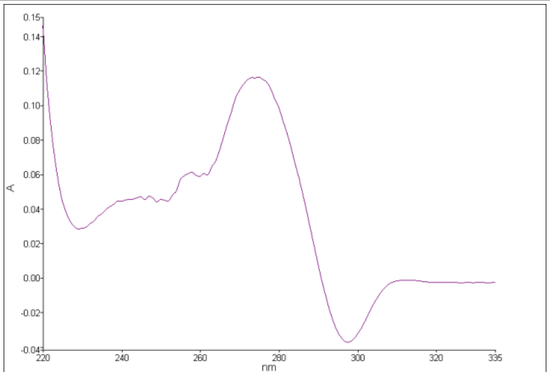
0.15
0.14
0.12
0.10
0.08
0.06
0.04
0.02
0.00
-0.02
-0.04
A
220
240
260
280
300
320
335
nm

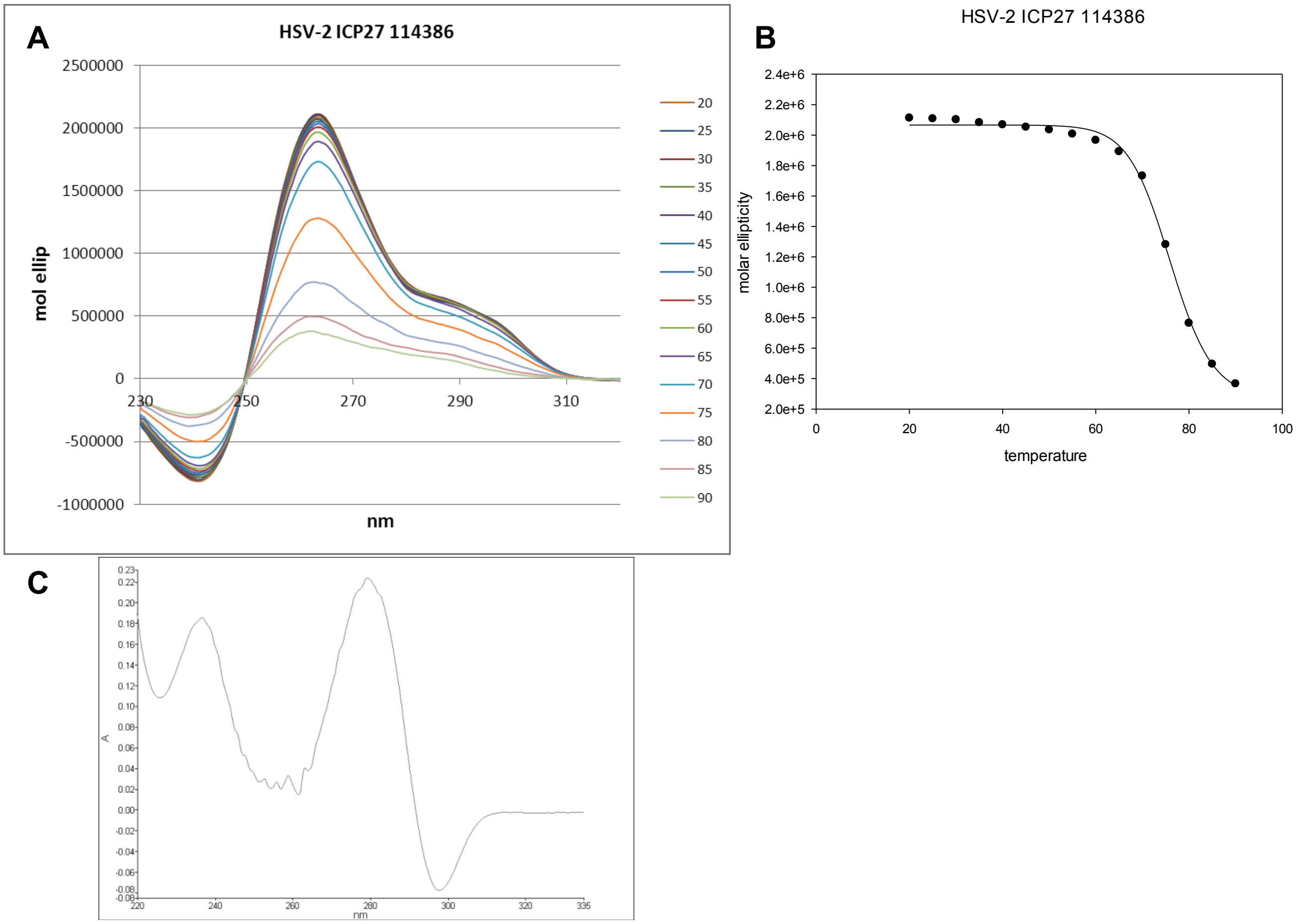
A
HSV-2 ICP27 114386
mol ellip
nm
2500000
2000000
1500000
1000000
500000
0
-500000
-1000000
230
250
270
290
310
20
25
30
35
40
45
50
55
60
65
70
75
80
85
90
B
HSV-2 ICP27 114386
molar ellipticity
temperature
2.4e+6
2.2e+6
2.0e+6
1.8e+6
1.6e+6
1.4e+6
1.2e+6
1.0e+6
8.0e+5
6.0e+5
4.0e+5
2.0e+5
0
20
40
60
80
100
C
A
nm
220
240
260
280
300
320
335

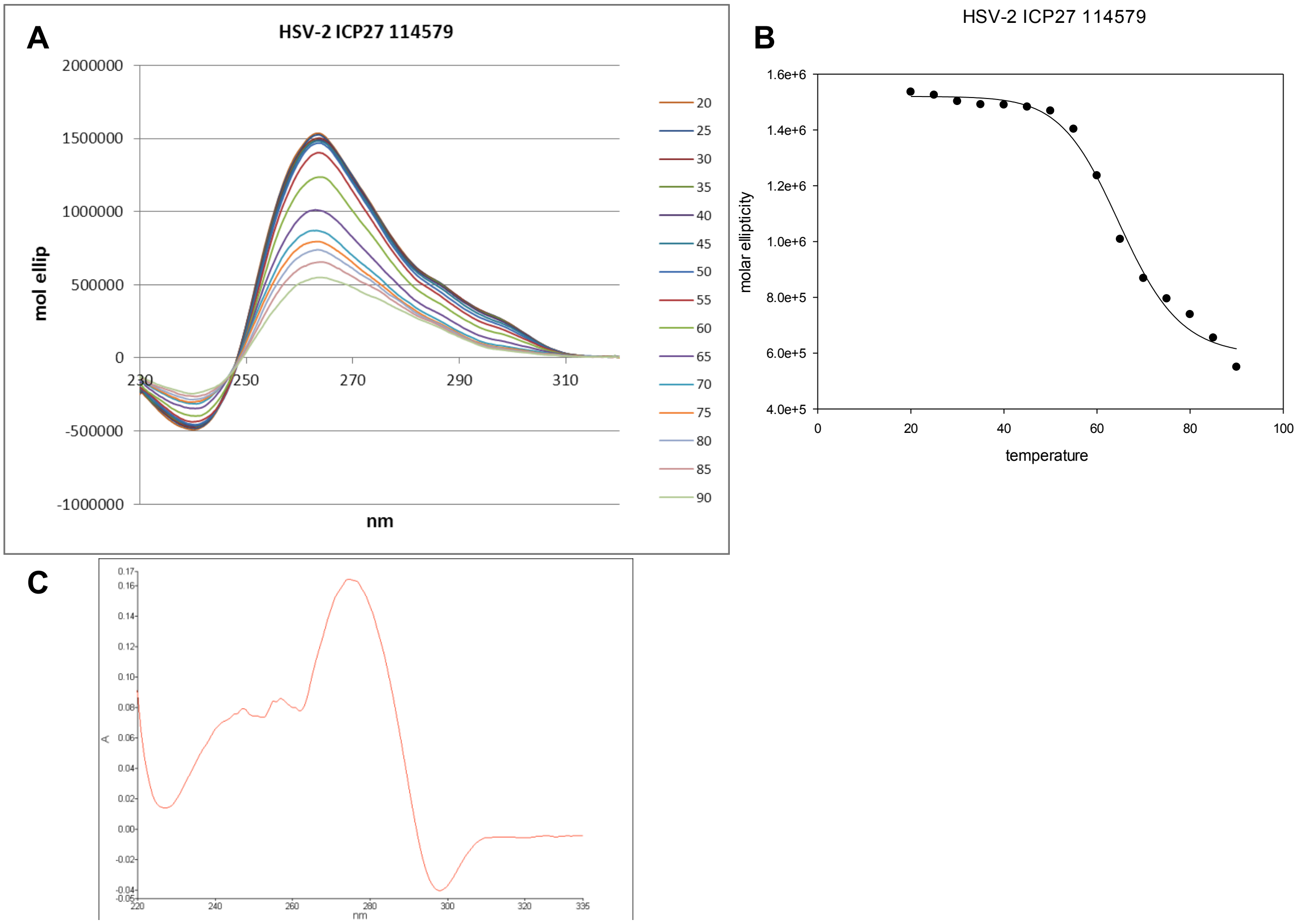
A
HSV-2 ICP27 114579
mol ellip
nm
2000000
1500000
1000000
500000
0
-500000
-1000000
230
250
270
290
310
20
25
30
35
40
45
50
55
60
65
70
75
80
85
90
B
HSV-2 ICP27 114579
molar ellipticity
temperature
1.6e+6
1.4e+6
1.2e+6
1.0e+6
8.0e+5
6.0e+5
4.0e+5
0
20
40
60
80
100
C
A
nm
220
240
260
280
300
320
335

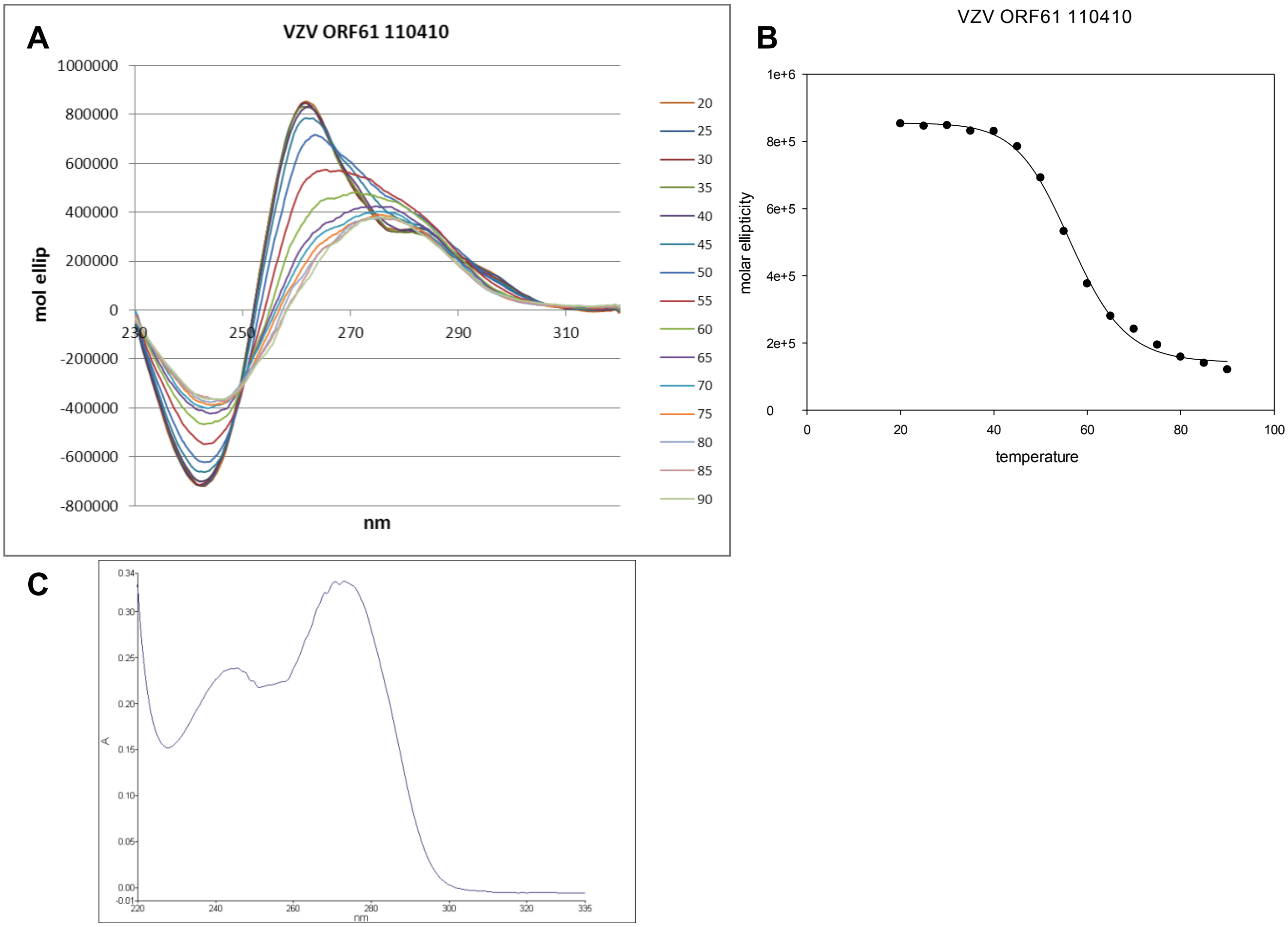
A
VZV ORF61 110410
1000000
800000
600000
400000
200000
0
-200000
-400000
-600000
-800000
mol ellip
230
250
270
290
310
nm
20
25
30
35
40
45
50
55
60
65
70
75
80
85
90
B
VZV ORF61 110410
1e+6
8e+5
6e+5
4e+5
2e+5
0
molar ellipticity
0
20
40
60
80
100
temperature
C
0.34
0.30
0.25
0.20
0.15
0.10
0.05
0.00
-0.01
A
220
240
260
280
300
320
335
nm

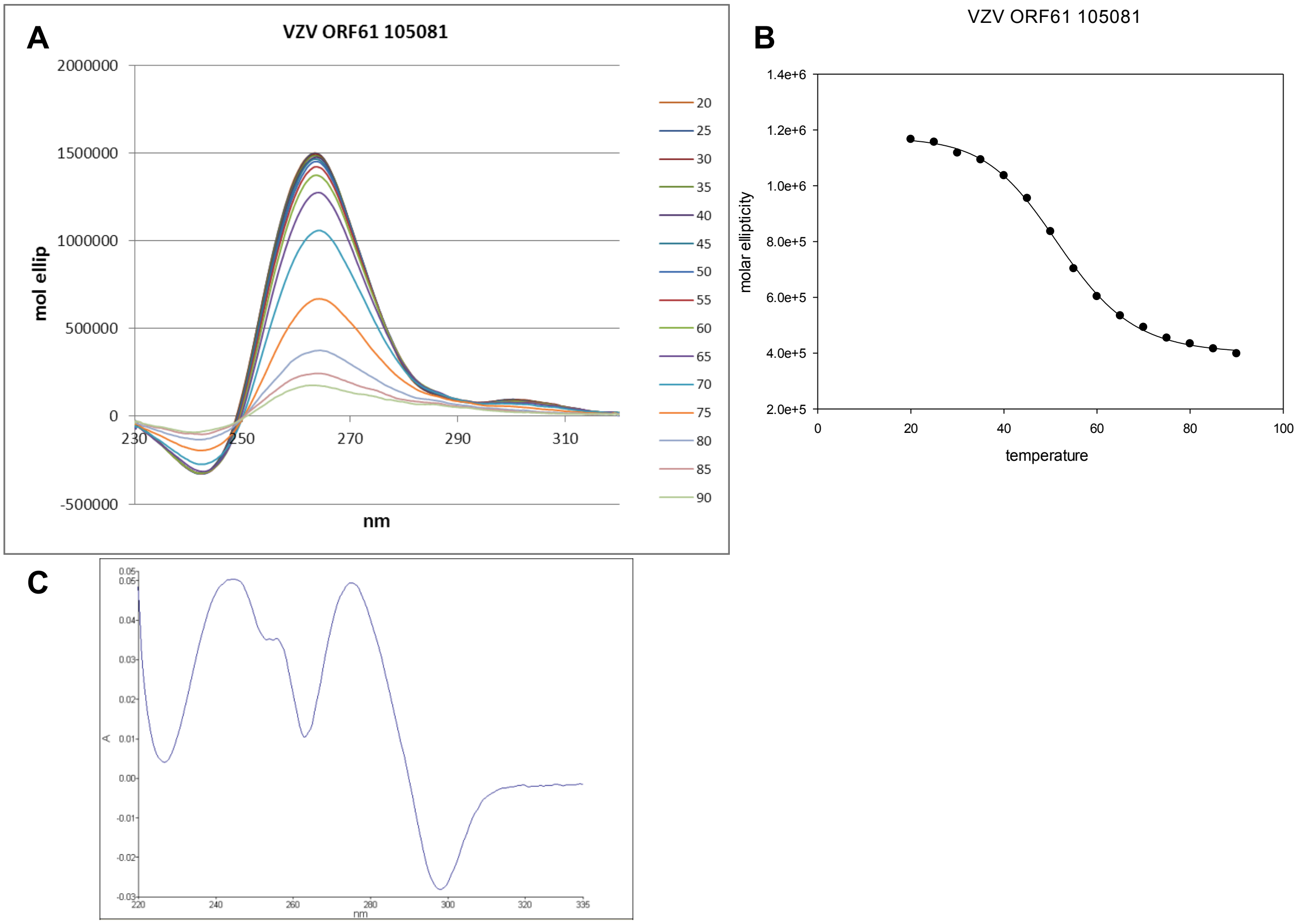
A
VZV ORF61 105081
mol ellip
2000000
1500000
1000000
500000
0
-500000
230
250
270
290
310
nm
20
25
30
35
40
45
50
55
60
65
70
75
80
85
90
B
VZV ORF61 105081
molar ellipticity
1.4e+6
1.2e+6
1.0e+6
8.0e+5
6.0e+5
4.0e+5
2.0e+5
0
20
40
60
80
100
temperature
C
A
nm
220
240
260
280
300
320
335

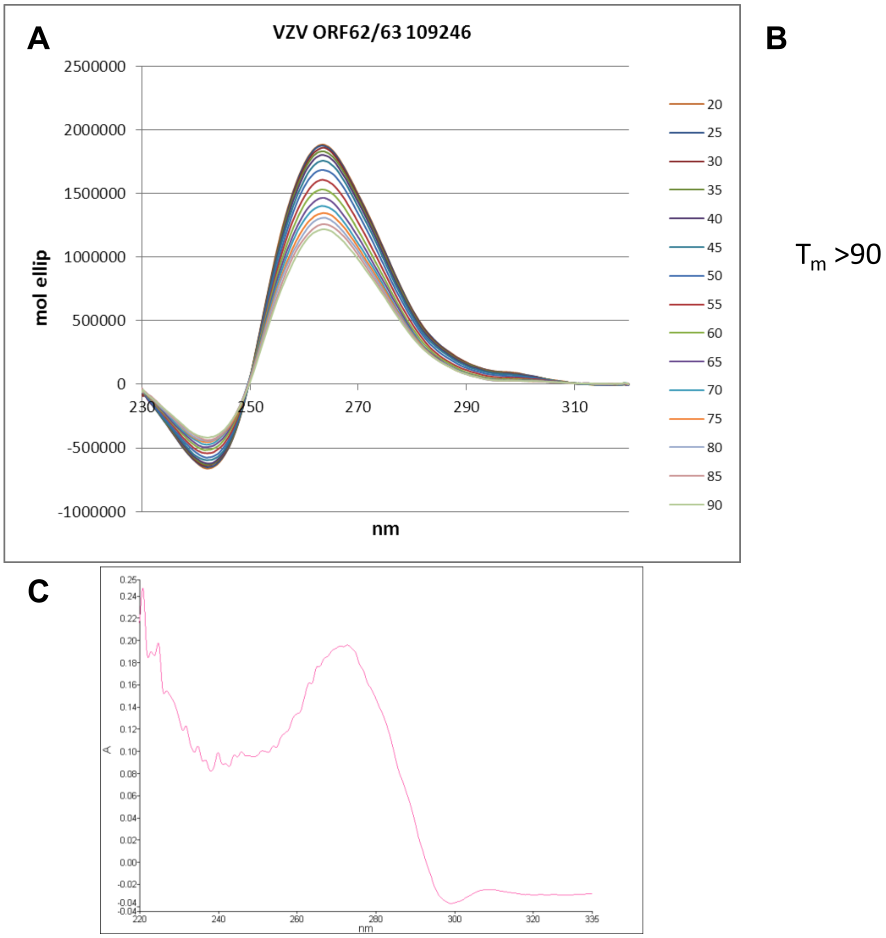

A
VZV ORF62/63 109246
mol ellip
nm
2500000
2000000
1500000
1000000
500000
0
-500000
-1000000
230
250
270
290
310
20
25
30
35
40
45
50
55
60
65
70
75
80
85
90
B
T_m >90
C
A
nm

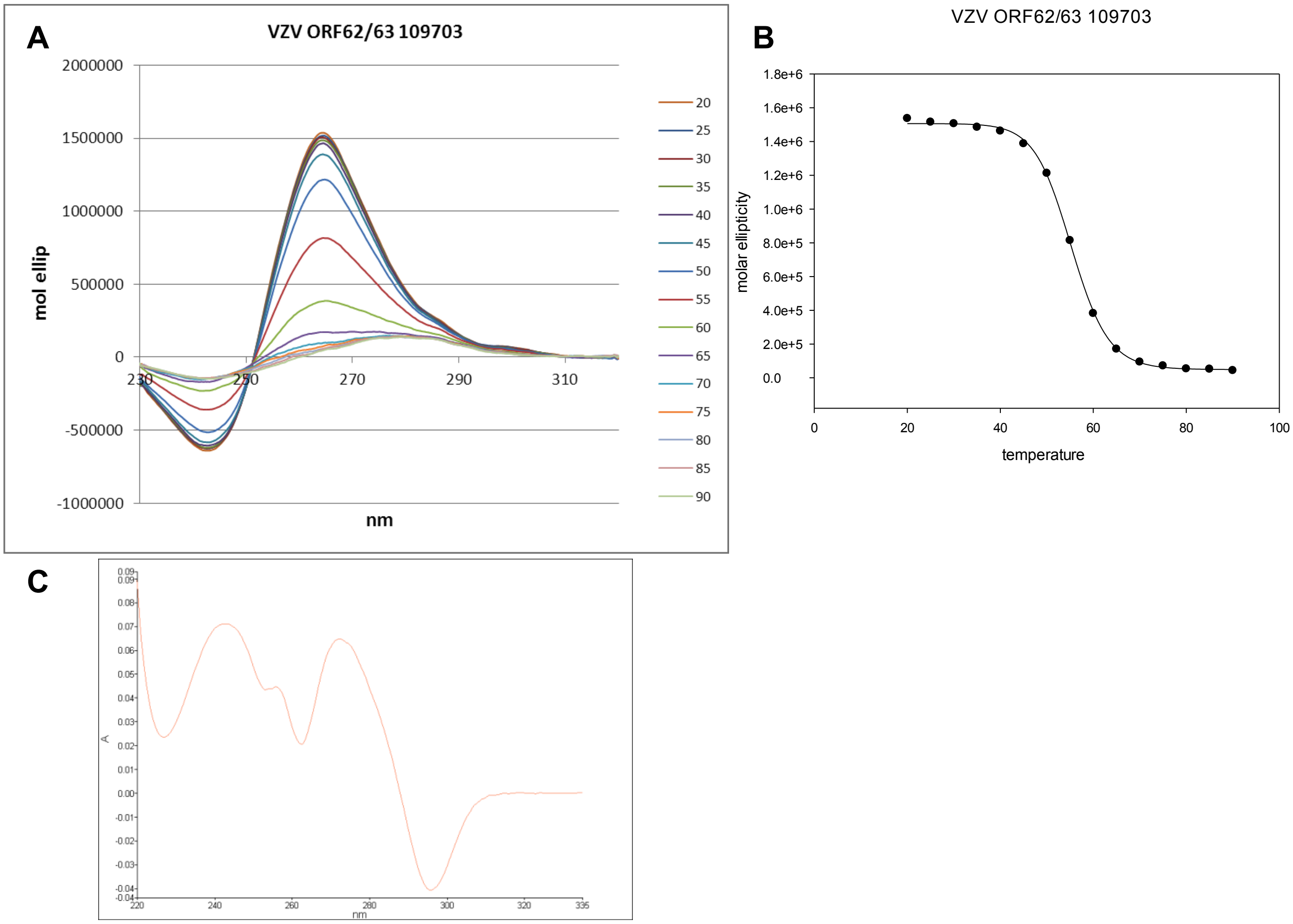

Table S1: oligonucleotides used in the Taq polymerase stop assay

Name	Sequence (5′-3′)
**ICP0**	TTTTTGGGGAGGGGAAAGGCGTGGGGTTTTTCTGCATATAAGCAGCTGCTTTTTGCC
**ICP27**	TTTTTGGGGCGGGGGCCCCGCCCGGGGGGCGGAACGAGGAGGGGTTTGGGTTTTTCTG CATATAAGCAGCTGCTTTTTGCC

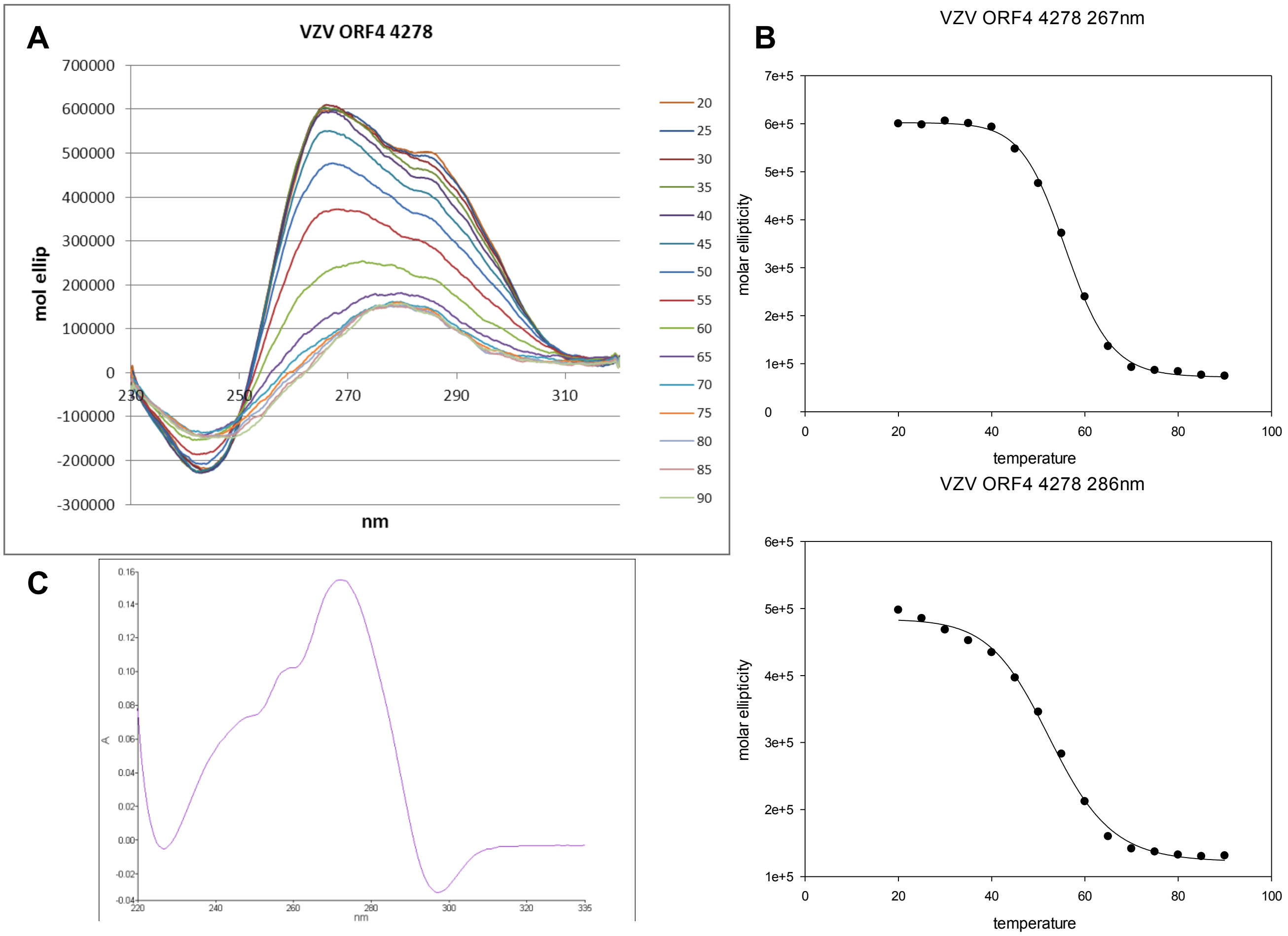

A
VZV ORF4 4278
mol ellip
nm
700000
600000
500000
400000
300000
200000
100000
0
-100000
-200000
-300000
230
250
270
290
310
20
25
30
35
40
45
50
55
60
65
70
75
80
85
90
B
VZV ORF4 4278 267nm
molar ellipticity
temperature
VZV ORF4 4278 286nm
molar ellipticity
temperature
C
nm
